# Sex-specific mitochondrial dysregulation and metformin response in Wilson disease

**DOI:** 10.1038/s41598-026-58720-7

**Published:** 2026-06-27

**Authors:** Valentina Medici, Noreene Shibata, Aldo Vorkapich, Jennifer Dang, Rida Mullah, Atena Tork, Isabel F. Snodgrass, Dohee Kim, John W. Newman, Zoe Lee Greenblatt, Huy-An Tran, Ieleen Li, Keren Sierra, Qixuan Gong, Cecilia Giulivi

**Affiliations:** 1 Department of Internal Medicine, Division of Gastroenterology and Hepatology, University of California Davis, Sacramento, CA USA; 2 Department of Molecular Biosciences, School of Veterinary Medicine, University of California Davis, Davis, CA USA; 3 West Coast Metabolomics Center, Genome Center, University of California Davis, Davis, CA USA; 4 United States Department of Agriculture, Agricultural Research Service, Western Human Nutrition Research Center, Davis, CA USA; 5 MIND Institute, University of California at Davis Medical Center, Sacramento, CA USA

**Keywords:** Copper toxicosis, Oxidative stress, Lipidomics, Glucocorticoids, Chelating agents, Liver, Mitochondrial ultrastructure, Diseases, Endocrinology, Medical research, Physiology

## Abstract

Wilson disease (WD) is an inherited disorder of copper metabolism characterized by hepatic copper accumulation, mitochondrial injury, and systemic metabolic dysfunction. Metformin is a widely used antihyperglycemic agent with known effects on mitochondrial metabolism and cellular redox state. We tested whether metformin modifies hepatic mitochondrial abnormalities in a mouse model of WD, stratified by sex. Adult male and female *Atp7b*^−/−^ mice were treated with metformin in drinking water (500 mg/kg/day) or water alone for 2 weeks. Liver mitochondria were evaluated by transmission electron microscopy and quantitative morphometry, respiratory chain enzyme activities, reactive oxygen species production, hepatic and mitochondrial copper and iron content, and targeted glucocorticoid and bile acid profiling. At baseline, female *Atp7b*^−/−^ mice exhibited greater mitochondrial ultrastructural injury, smaller mitochondrial size, and higher hepatic and mitochondrial copper and iron levels compared with males. Metformin exposure was higher in females and was associated with marked changes in mitochondrial morphology, including increased size and more regularized structural features, along with alterations in respiratory enzyme activities, reduced oxidative stress, and changes in glucocorticoid and bile acid profiles. In contrast, males showed more limited structural and biochemical changes following treatment. These findings identify sex as an important determinant of mitochondrial phenotype and response to metformin in WD and support further investigation of sex-informed therapeutic strategies.

**Supplementary Information:**

The online version contains supplementary material available at https://doi.org/10.1038/s41598-026-58720-7.

## Introduction

Wilson disease (WD) is an autosomal genetic disorder of copper metabolism caused by pathogenic variants in the *ATP7B* gene on chromosome 13q14, resulting in impaired biliary copper excretion and progressive hepatic copper accumulation^1^. When hepatic storage capacity is exceeded, copper enters the circulation and deposits in extrahepatic tissues, producing multisystem disease with hepatic, neurologic, and psychiatric manifestations. Although WD is not sex-linked, clinically relevant sex differences are well documented. Male patients are more often diagnosed later in life and present predominantly with neuropsychiatric features. In contrast, females more frequently develop earlier and more severe liver disease, including acute liver failure and other hepatic phenotypes^2,3^.

The liver is a major site of copper accumulation in WD and in experimental copper overload, with hepatic copper levels up to 18-fold higher than in noncarriers^4,5^. Within hepatocytes, mitochondria are a major intracellular reservoir for copper, accounting for approximately 35% of total hepatic copper in WD compared with 16% in noncarriers^6,7^. Excess mitochondrial copper is associated with marked structural and functional abnormalities, including swelling, elongation, matrix condensation, and cristae disruption^8,9^. Consistent with these ultrastructural changes, WD is characterized by impaired oxidative phosphorylation, reduced aconitase activity, decreased expression of respiratory Complex IV subunits, and mitochondrial DNA depletion^5,10,11^. Similar defects, accompanied by increased oxidative stress and altered lipid metabolism, have been demonstrated in multiple WD mouse models^11–15^. More recently, copper-induced mitochondrial injury has been linked to cuproptosis, a regulated form of cell death associated with mitochondrial protein aggregation and bioenergetic failure^16,17^. Together, these findings highlight mitochondrial copper dysregulation as a central driver of hepatic injury in WD, with potential implications for therapeutic response^18^.

Current first-line therapy for WD relies on copper chelators, including D-penicillamine, which enhances urinary copper excretion and improve long-term outcomes when initiated early^19^, or trientine, which acts both by reducing intestinal copper absorption and increasing urinary copper excretion^20^. However, clinical presentation varies by sex^2^, and conventional chelators appear to have limited impact on mitochondrial copper pools. Methanobactin SB2 (ARBM101), a high-affinity copper-binding peptide, has shown marked efficacy in reducing hepatic and mitochondrial copper and restoring mitochondrial structure and function in rodent models^21,22^. Despite these promising results, such agents remain experimental, and their accessibility and clinical implementation are uncertain.

Metformin, a widely used oral antihyperglycemic agent, forms high-affinity complexes with transition metals, including copper, through its bidentate biguanide structure^23^. Although metformin’s glucose-lowering effects have been attributed to inhibition of hepatic gluconeogenesis via mitochondrial energy stress and activation of AMP-activated protein kinase (AMPK)^24,25^, its metal-binding properties have received comparatively little attention. Notably, metformin activates AMPK only in the presence of intracellular copper^26^, and biguanides can alter the redox state of mitochondrial copper bound to proteins^27,28^. Mitochondria-targeted metformin analogs and metformin itself have also shown synergistic effects with iron chelators in experimental cancer models, further supporting a role for metal-dependent mitochondrial actions^29^.

Given growing epidemiologic and experimental evidence supporting the repurposing of metformin beyond diabetes^30–32^, its established safety and pharmacokinetics^31^, its ability to accumulate in mitochondria as a weak cation^33^, and its inclusion on the World Health Organization Model List of Essential Medicines^34^, we hypothesized that metformin could mitigate hepatic mitochondrial injury in WD by modulating copper-dependent mitochondrial dysfunction. Importantly, while mitochondrial abnormalities are a hallmark of WD and correlate with disease severity, quantitative analyses of hepatic mitochondrial morphology in WD are limited, and sex-specific responses to metformin have not been examined.

To address these gaps, we treated male and female *Atp7b*^*−/−*^ global knockout mice on a C57BL/6 background, an established model of WD, with metformin administered in drinking water (approximately 500 mg/kg/day) for 2 weeks. This dosing strategy has been widely used in experimental models and produces therapeutic plasma concentrations comparable to those observed in humans^35,36^. We performed an integrated analysis of hepatic and mitochondrial copper and iron content, mitochondrial ultrastructure and bioenergetic function, oxidative stress, and lipid mediator profiles, including glucocorticoids, sex hormones, oxylipins, and bile acids, before and after treatment. This approach allowed us to define sex-specific mitochondrial and metabolic responses to metformin in WD and to evaluate its potential as a precision adjunctive therapy targeting hepatic mitochondrial dysfunction.

## Results

### Metformin exposure and whole-body metabolic responses differ by sex in *Atp7b*^*−/−*^ mice

**Fig. 1:**
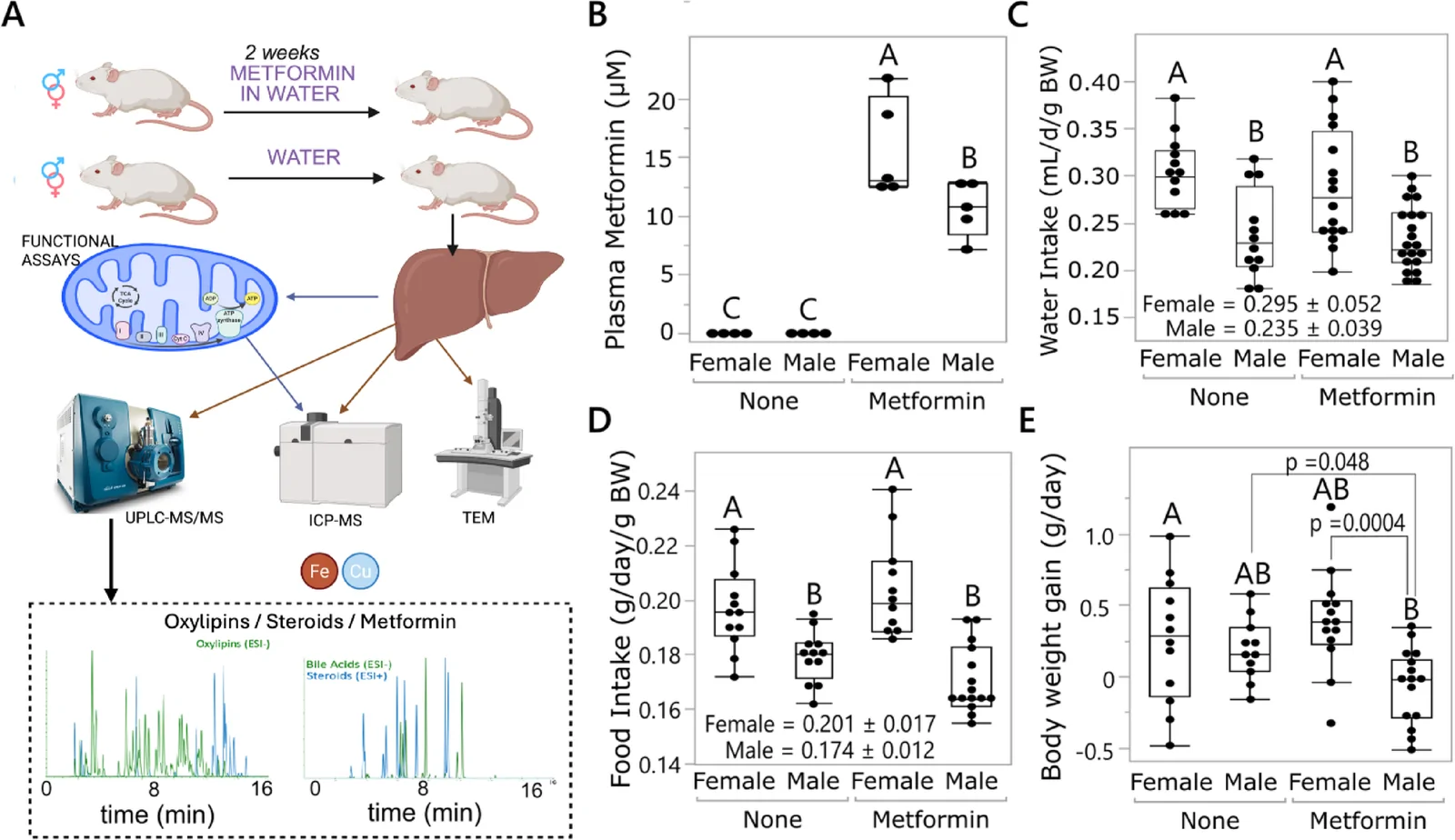
Study design, metformin exposure, and whole-body metabolic parameters in Atp7b^−/−^ mice. (**A**) Experimental design. Adult female and male *Atp7b*^−/−^ mice (WD model) were treated with normal water (untreated *Atp7b*^−/−^ mice; *n* = 4 M/4F) or metformin (*n* = 5 M/5F) in drinking water for 2 weeks. At study completion, livers were collected for transmission electron microscopy (TEM), mitochondrial functional analyses, iron and copper quantification in whole liver and isolated mitochondria, and lipid mediator, steroid, and bile acid profiling. Schematic created with BioRender.com. (**B**) Plasma metformin concentrations measured by mass spectrometry at the end of the 2-week treatment period. (**C**) Water consumption normalized to body weight (mL/day/g body weight). (**D**) Food intake normalized to body weight (g/day/g body weight). (E) Body weight gain expressed as percentage change from baseline. Data are presented as mean ± SD. Statistical analyses were performed using two-way ANOVA followed by Tukey’s HSD post hoc test. Groups sharing the same letter are not significantly different.

To assess whether metformin exerts sex-dependent effects in Wilson disease (WD), adult *Atp7b*^−/−^ mice (12–14 weeks old), a genetic model of WD, were treated with metformin administered via drinking water for 2 weeks (*n* = 10; 5 females and 5 males; 500 mg/kg/day). Untreated *Atp7b*^*−/−*^ mice (*n* = 8; 4 females and 4 males) were maintained under identical conditions without metformin supplementation (Fig. 1A). Liver tissue was collected for transmission electron microscopy (TEM), assessment of mitochondrial complex activities, quantification of iron and copper by ICP-MS, and lipid mediator profiling by UPLC-MS/MS. Metal and lipid mediator analyses were performed in both whole liver homogenates and isolated mitochondria. Based on measured water intake, estimated metformin exposure remained within the intended dosing range for both sexes.

Plasma metformin levels showed a significant treatment effect, with higher concentrations in females than in males (Fig. 1B). The higher circulating metformin levels observed in females may partly reflect increased drug intake secondary to greater water consumption. Indeed, daily water intake normalized to body weight was consistently higher in females than in males [mean ± SD: 0.295 ± 0.052 mL x (d x g body weight)^−1^ in females and 0.235 ± 0.039 mL x (d x g body weight)^−1^ in males], regardless of treatment (Fig. 1C). However, the magnitude of this difference (~ 1.2-fold) was smaller than the observed increase in plasma metformin concentrations (~ 1.5-fold), suggesting that differences in water consumption alone do not fully explain the sex-dependent exposure and are consistent with sex-specific differences in metformin pharmacokinetics.

Daily food intake, normalized to body weight, was also higher in females than in males [mean ± SD: 0.201 ± 0.017 g x (d x g body weight)^−1^ in females and 0.174 ± 0.012 g x (d x g body weight)^−1^ in males] and was unaffected by metformin treatment (Fig. 1D). Analysis of body weight gain—calculated as the percentage change relative to day 1—revealed greater weight gain in untreated males compared with metformin-treated males (*P* = 0.048; Fig. 1E). In contrast, metformin-treated females gained significantly more weight than treated males (*P* = 0.004; Fig. 1E), despite no corresponding increase in food intake, suggesting altered metabolic efficiency rather than hyperphagia.

Collectively, these data show that plasma metformin concentrations were undetectable at baseline and increased robustly following treatment in both sexes. Notably, females achieved significantly higher circulating metformin levels than males despite comparable dosing. While females consumed slightly more water, this difference did not fully account for the increased exposure, suggesting that factors beyond water consumption may contribute to sex-dependent differences in metformin exposure. Downstream hepatic and mitochondrial endpoints were therefore examined to determine whether these exposure differences translated into functional metabolic consequences.

### Severe mitochondrial ultrastructural injury is a core feature of *Atp7b*^*−/−*^ livers

Given that mitochondrial structural alterations and related pathways represent early indicators of liver injury in asymptomatic WD^8^, this approach enabled evaluation of sex differences^26^ and metformin response within a defined ultrastructural framework. The number of TEM images and mitochondrial counts per mouse, stratified by sex and treatment, were summarized under Supplementary Table 1.

Consistent with previous reports describing mitochondrial ultrastructural abnormalities in WD^37^, liver TEM imaging revealed mitochondria with aberrant morphology in untreated *Atp7b*^−/−^ mice of both sexes (Supplementary Fig. 1). Notably, and to our knowledge and for the first time in this model, we observed mitochondrial protrusions that occasionally gave rise to small, electron-dense vesicles derived from mitochondria (mitochondria-derived vesicles; MDVs; Supplementary Fig. 1). Additional ultrastructural abnormalities previously reported by others^38^ were also evident and included intramitochondrial vacuoles, disruptions of the mitochondrial outer membrane, and enlargement of the intermembrane space. Defects of the inner membrane were characterized by cristae loss and increased intercristae spacing, whereas matrix abnormalities included electron-dense inclusions and paracrystalline structures (Supplementary Fig. 2). Evidence of broader organelle stress was also present, including autophagosomes engulfing mitochondria, lysosomes containing dark deposits consistent with metal accumulation, abundant cytosolic lipid vacuoles, and relatively uniform dilatation of endoplasmic reticulum cisternae (Supplementary Fig. 1). Paracrystalline inclusions were more prevalent in metformin-treated *Atp7b*^−/−^ mice of both sexes (Supplementary Fig. 2). Together, these qualitative observations indicate substantial cellular stress associated with copper accumulation and reveal ultrastructural features consistent with increased MDV formation, autophagic structures involving mitochondria, lysosomal accumulation of electron-dense material, and widespread organelle remodeling.

**Fig. 2:**
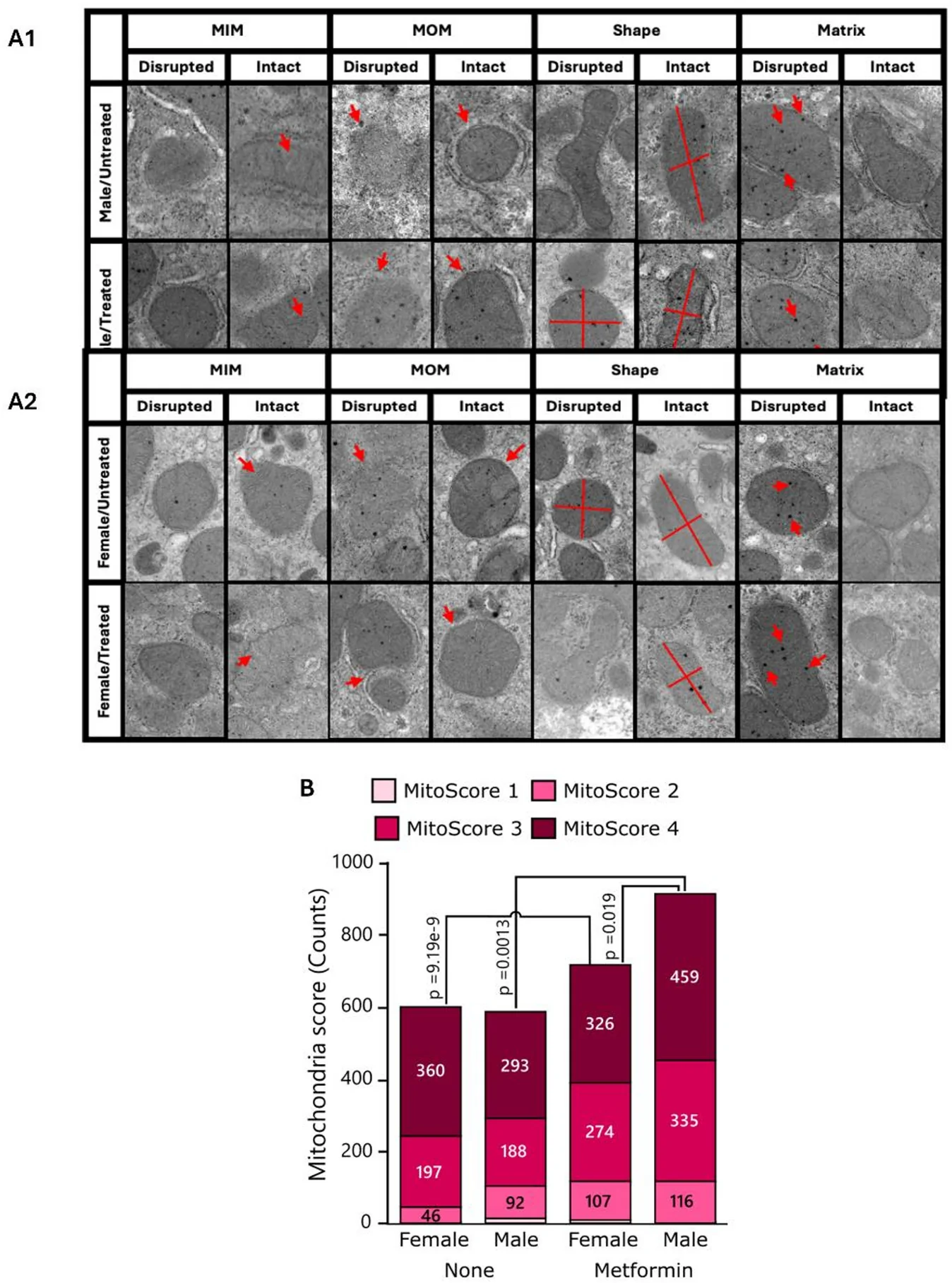
Quantitative assessment of liver mitochondrial ultrastructural injury in Atp7b^−/−^ mice. (**A**) Schematic representation of the mitochondrial injury scoring system based on transmission electron microscopy, shown by sex and treatment group (Panel A1: male untreated and male treated, Panel A2: female untreated and female treated). Mitochondria were scored using four ultrastructural criteria: matrix appearance, overall shape, mitochondrial outer membrane (MOM) integrity, and mitochondrial inner membrane (MIM) organization. For each criterion, a score of 0 indicated preserved structure and a score of 1 indicated abnormality. Individual scores were summed to generate a composite mitochondrial injury score ranging from 0 (structurally intact) to 4 (severely damaged). (**B**) Quantitative analysis of mitochondrial injury scores by sex and metformin treatment. MitoScore values ranged from 0 (best) to 4 (worst) based on matrix appearance, overall shape, mitochondrial outer membrane integrity, and mitochondrial inner membrane organization. Data were analyzed using two-way ANOVA followed by Tukey’s HSD post hoc test.

To enable systematic comparison of mitochondrial ultrastructure across groups, qualitative TEM observations were converted into a semiquantitative scoring system. Mitochondria were evaluated based on four criteria: matrix appearance, overall shape, mitochondrial outer membrane (MOM) integrity, and mitochondrial inner membrane (MIM) organization, including cristae structure. Each parameter was assigned a score of 0 (intact) or 1 (disrupted), and values were summed to generate a composite injury score ranging from 0 (structurally intact) to 4 (severely damaged). Representative images are shown by sex and treatment group (Fig. 2A1,A2). This approach facilitated the detection of group-level differences that were not readily apparent by qualitative inspection alone. A total of 86 images from untreated mice (1191 mitochondria) and 110 images from metformin-treated mice (1633 mitochondria) were analyzed. The 2824 mitochondria were analyzed across four groups defined by sex (female, male) and treatment condition (untreated, treated; Fig. 2B). Scores ranged from 1 to 4, with lower scores indicating better outcomes. At baseline (untreated condition), there was a statistically significant difference in score distributions between females and males (χ² test of independence, *p* < 0.001). Females exhibited slightly higher scores (mean = 3.52, median = 4) compared to males (mean = 3.31, median = 4), indicating worse outcomes in females prior to treatment. Following treatment, a sex-specific effect was observed. In females, treatment was associated with a shift toward lower scores, with the mean decreasing from 3.52 to 3.29 and the median shifting from 4 to 3, indicating improved outcomes. In contrast, males did not demonstrate improvement with treatment; mean scores increased slightly from 3.31 to 3.38, and the median remained unchanged at 4. A comparison of score distributions across all groups using a chi-square test confirmed a statistically significant difference (χ²(9) ≈ 130, *p* < 0.000001), although the overall effect size was small (Cramér’s V ≈ 0.12). Pairwise comparisons showed that untreated males differed significantly from all other groups, whereas treated females differed significantly from untreated females, consistent with a treatment effect in females but not in males. These findings indicate a significant interaction between sex and treatment condition, with treatment improving outcomes in females but not in males.

### Metformin does not reduce hepatic copper burden but alters mitochondrial metal deposition

To evaluate the characteristic metal accumulation associated with WD, TEM imaging revealed abundant electron-dense deposits within lysosomes, mitochondria, and the cytosol of hepatocytes, consistent with previous reports of WD and copper toxicosis^39,40^ and likely representing copper and iron accumulation^41^. These deposits were observed across sexes and treatment groups, confirming advanced metal dysregulation in the *Atp7b*^−/−^ liver. Mixed-effects modeling with mouse ID included as a random factor and sex and treatment as fixed factors revealed no significant differences in the number of mitochondrial electron-dense deposits across groups or sexes (number of liver mitochondrial deposits/mouse: untreated females: 310 ± 52; metformin-treated females: 285 ± 46; untreated males: 363 ± 52; metformin-treated males: 402 ± 46). In contrast, deposit diameter showed a modest treatment effect, with metformin associated with a small increase in average diameter (6.5%; DF = 1, DFDen = 14, F = 5.18, *P* = 0.039).

**Fig. 3:**
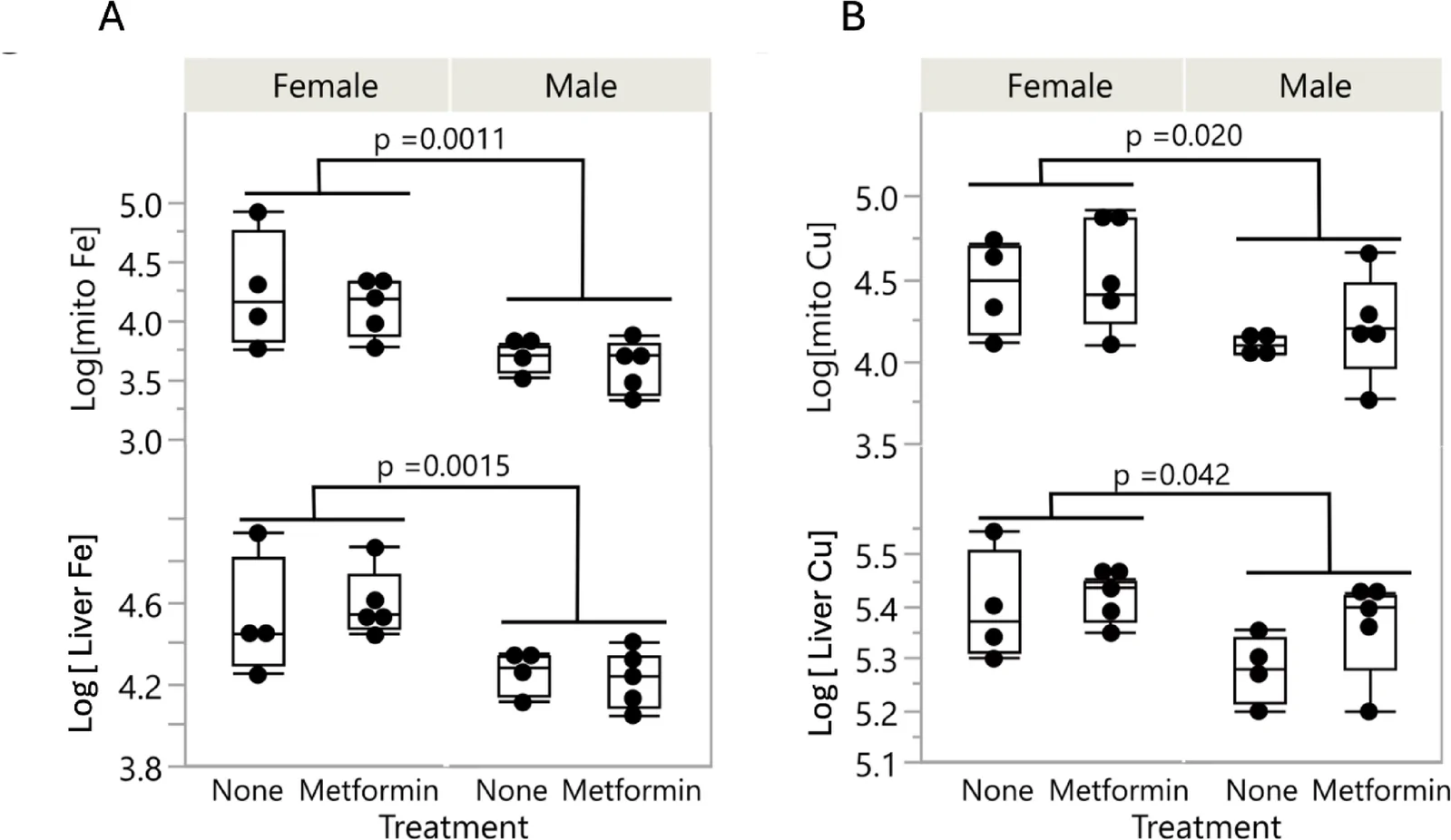
Mitochondrial electron-dense deposits and hepatic copper and iron content in Atp7b^−/−^ mice. (**A**) Hepatic iron content expressed as µg Fe/mg wet liver weight (bottom) and as total ng Fe per mitochondrial mass (top), shown for females and males with and without metformin treatment. (**B**) Hepatic copper content expressed as µg Cu/mg wet liver weight (bottom) and as total ng Cu per mitochondrial mass (top), shown for females and males with and without metformin treatment. Data were log-transformed and analyzed by ANOVA followed by least-squares means contrast post hoc testing. Significant effects are reported where indicated (*P* < 0.05).

Because electron-dense inclusions in WD may arise from copper and/or iron accumulation, hepatic and mitochondrial metal content was quantified using ICP-MS in age- and sex-matched untreated and metformin-treated *Atp7b*^−/−^ mice (Supplementary Table 2; Methods). This analysis served both to validate the murine WD model and to determine whether ultrastructural metal deposition correlated with bulk tissue metal levels. Mitochondrial and hepatic iron concentrations were comparable between metformin-treated and untreated *Atp7b*^−/−^ mice (Fig. 3A). However, a significant sex interaction was observed, with females exhibiting higher iron content in both mitochondria and whole liver compared with males (Log[mito Fe]: *P* = 0.0011; Log[Liver Fe]: *P* = 0.0015). A similar pattern was observed for copper concentrations in mitochondria and liver tissue (Log[mito Cu]: *P* = 0.020; Log[Liver Cu]: *P* = 0.042; Fig. 3B). Importantly, metformin treatment did not significantly alter total hepatic or mitochondrial copper or iron levels in either sex, indicating that drug exposure did not reduce overall metal burden.

In summary, electron microscopy demonstrated extensive electron-dense inclusions in hepatocytes localized to lysosomes, cytosol, and mitochondria, consistent with pathological metal accumulation in WD (Fig. 3). Quantitative analysis revealed that metformin increased the number of intramitochondrial deposits in both sexes, with a greater relative increase observed in females. Deposit size increased modestly with treatment but did not differ by sex. Importantly, ICP-MS analysis confirmed that metformin did not reduce total hepatic or mitochondrial copper or iron content. Instead, females consistently exhibited higher metal levels than males across compartments, independent of treatment. Together, these findings indicate that metformin did not decrease total hepatic or mitochondrial metal content but instead changed mitochondrial ultrastructure and metal handling in the presence of ongoing copper overload.

### Quantitative morphometry reveals sex-dependent mitochondrial remodeling with metformin

Because the semiquantitative mitochondrial injury scores had a small overall effect size and ultrastructural differences were not readily apparent on qualitative inspection alone, we performed a more in-depth, unbiased quantitative morphometric analysis of thousands of mitochondria (Supplementary Table 3; Methods). This approach enabled objective detection of subtle but biologically meaningful sex- and treatment-dependent differences.

**Fig. 4:**
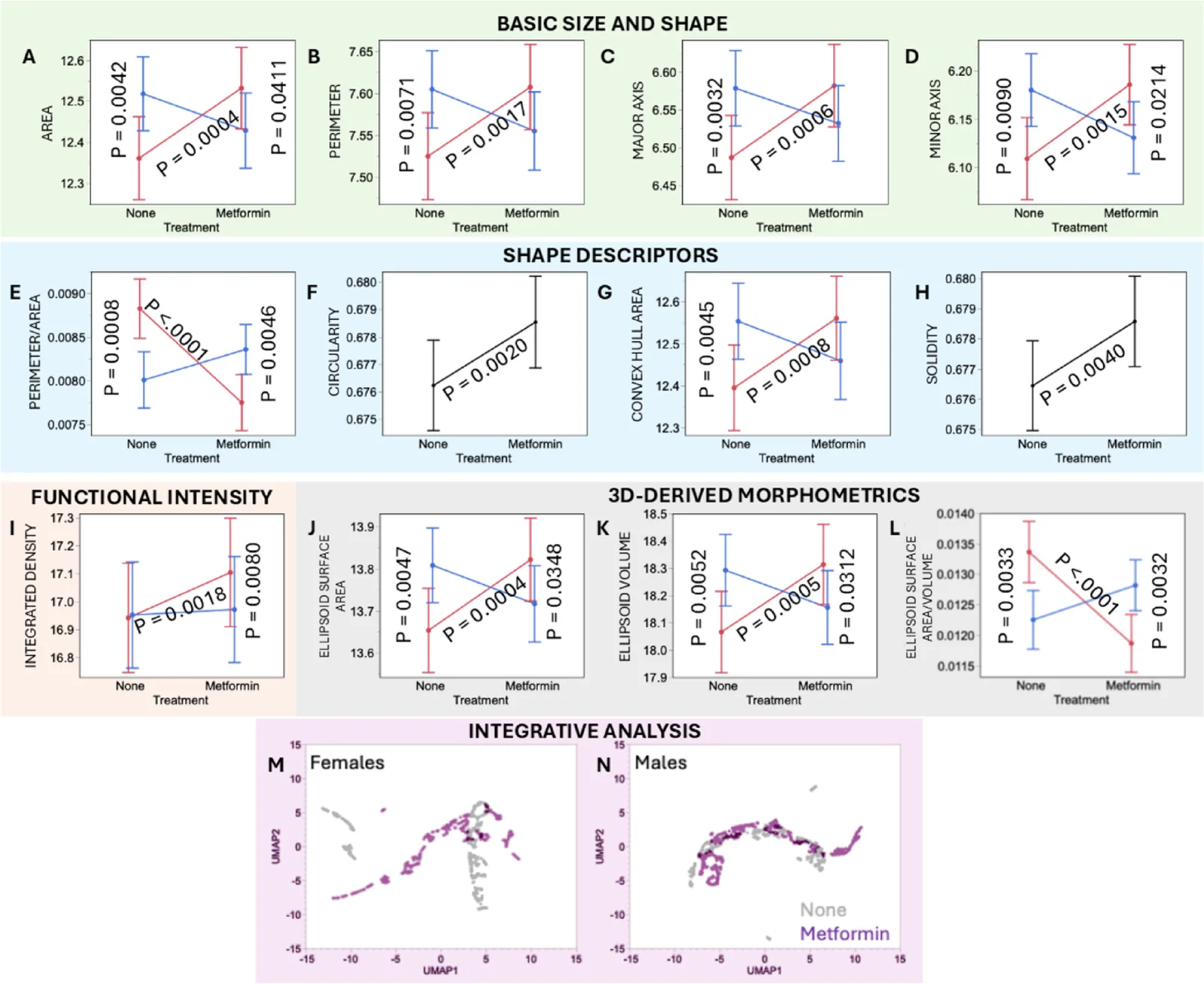
Sex-dependent mitochondrial morphometry and structural remodeling in Atp7b^−/−^ mice. Quantitative morphometric parameters of liver mitochondria were analyzed across sex and metformin treatment. Male mitochondria are shown in blue and female mitochondria in red. Data were analyzed for effects of sex, treatment, and sex × treatment interaction using two-way ANOVA followed by Tukey’s HSD post hoc test. Least-squares means are shown. Significant effects are reported where indicated (*P* < 0.05). (**A**–**D**) Mitochondrial area, perimeter, major axis, and minor axis. (**E**–**H**) Perimeter-to-area ratio, circularity, convex hull area, and solidity. (**I**–**L**) Integrated density, estimated ellipsoid surface area, ellipsoid volume, and ellipsoid surface area-to-volume ratio. (**M**–**N**) Uniform Manifold Approximation and Projection (UMAP) based on all measured morphometric parameters, illustrating multivariate structural differences by sex and treatment.

Liver mitochondria were segmented from TEM images, and morphometric parameters were quantified using ImageJ, including area, perimeter, major and minor axes, solidity, circularity, and convex hull, along with additional shape descriptors (Fig. 4). All measurements were compiled at the level of individual mitochondria and analyzed using a mixed-effects model, with sex, treatment, and their interaction specified as fixed effects and image number included as a random effect. Post hoc comparisons were performed using Tukey’s HSD test. This modeling strategy accounts for within-image correlation and unequal sampling across images.

At baseline, female WD mice exhibited significantly smaller hepatic mitochondria than males, with reductions in cross-sectional area, perimeter, major and minor axes, convex hull area, estimated surface area, and volume. In contrast, parameters reflecting overall shape—including aspect ratio, convexity, circularity, elongation, form factor, and solidity—did not differ between sexes at baseline, indicating that mitochondrial shape was preserved despite reduced size. Following metformin treatment, liver mitochondria from female WD mice exhibited significantly larger areas, perimeters, major and minor axes (with unchanged aspect ratios), convex hull areas, integrated densities, estimated surface areas, and volumes compared with those from treated males. Relative to treated males, treated females also exhibited lower perimeter-to-area ratios and surface-to-volume ratios, indicating reduced boundary complexity relative to mitochondrial size. These changes are consistent with reduced boundary complexity relative to mitochondrial size. Regardless of sex, metformin treatment increased mitochondrial circularity and solidity, as reflected by fewer irregular boundaries and improved structural coherence (Fig. 4A-L).

To capture higher-order structural relationships, we applied Uniform Manifold Approximation and Projection (UMAP) analysis to the full set of morphometric parameters (Fig. 4M, N). This multivariate analysis revealed clear separation of female mitochondria by treatment condition, whereas male mitochondria largely overlapped across treatment groups. Thus, multivariate remodeling was more prominent in females than in males. These findings indicate that female liver mitochondria exhibit broader morphometric changes in response to metformin with coordinated changes across multiple structural dimensions, while male mitochondria show comparatively modest treatment-associated remodeling.

In sum, at baseline, female *Atp7b*^−/−^ mice exhibited significantly smaller hepatic mitochondria than males, despite preservation of overall shape. Metformin induced pronounced sex-dependent mitochondrial remodeling. In females, mitochondria became larger, rounder, and structurally smoother, as reflected by increases in area and volume and reductions in surface-to-volume and perimeter-to-area ratios. In contrast, male mitochondria exhibited limited size expansion and less consistent structural refinement. Integration of multiple morphometric features through dimensionality reduction further revealed clear segregation of female mitochondria following metformin treatment, whereas male mitochondria remained largely overlapping across conditions. Collectively, these findings demonstrate that while semi-quantitative injury scoring detects only modest group differences, quantitative morphometric analysis revealed substantial sex-dependent mitochondrial remodeling, with female hepatic mitochondria exhibiting greater structural plasticity in response to metformin under conditions of copper-induced stress.

### Metformin shifts morphometry-based mitochondrial dynamics classifications toward fusion predominantly in females

Given the observed sex-dependent changes in mitochondrial size and geometry, we next assessed whether metformin altered mitochondrial dynamics. Using validated morphometric thresholds associated with mitochondrial fission and fusion states^38^, individual mitochondria were classified based on structural features. This morphometry-based approach provides an indirect, morphology-based proxy of mitochondrial dynamics, allowing quantitative assessment of structural states associated with fission- or fusion-like morphology at the whole-cell level, but does not directly measure the molecular fission–fusion machinery. Based on prior work demonstrating that increased mitochondrial perimeter (normalized to area) and increased solidity are positively associated with mitochondria poised to undergo fission and fusion, respectively^38^, we evaluated the distributions of the perimeter-to-area ratio and solidity.

**Fig. 5:**
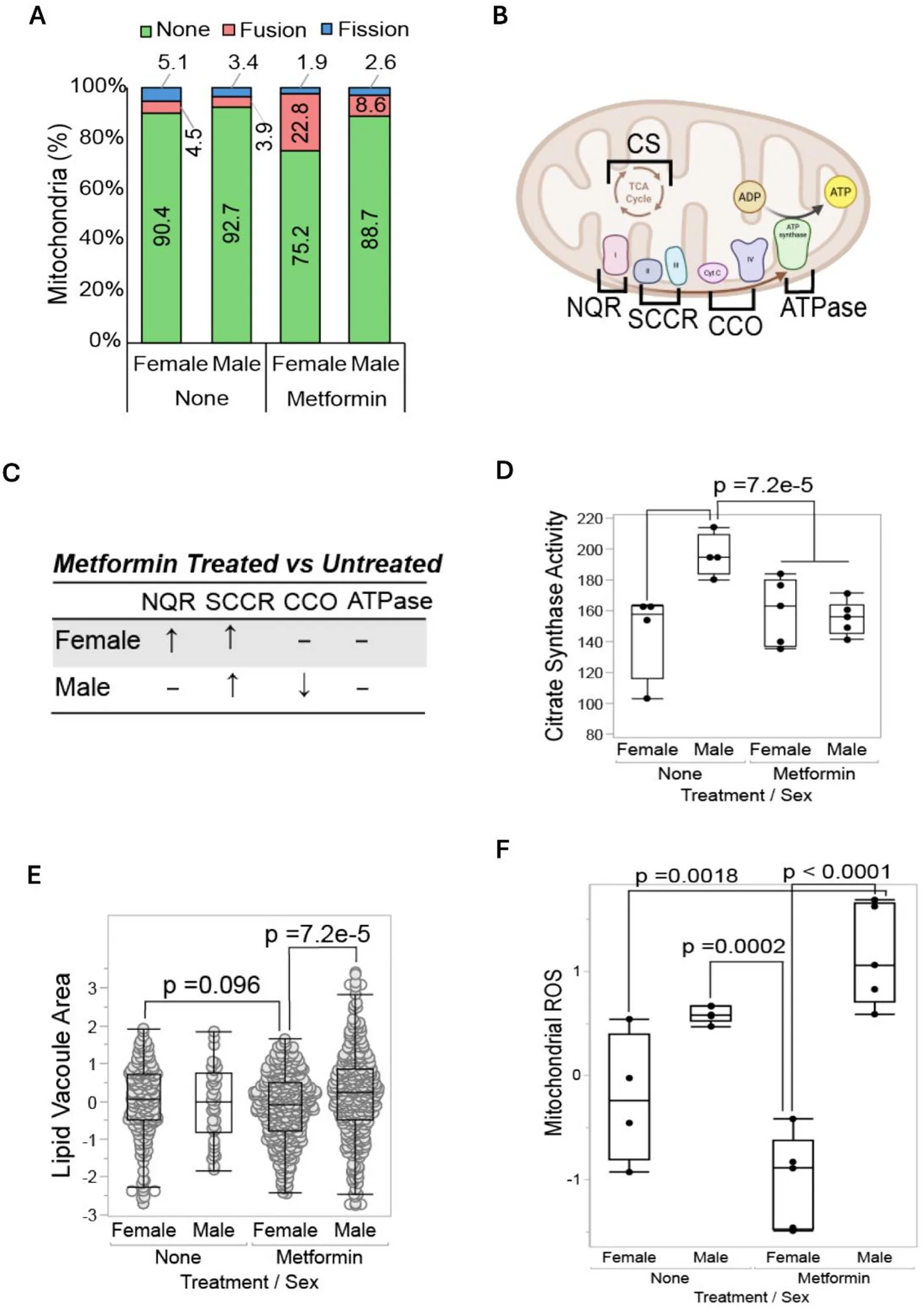
Sex-dependent effects of metformin on mitochondrial dynamics, bioenergetics, lipid accumulation, and oxidative stress in Atp7b^−/−^ mice (**A**) Contingency plot showing the proportion of mitochondria classified as exhibiting no dynamic event (“none”), high fission risk (“fission”), or high fusion risk (“fusion”), stratified by sex and metformin treatment. (**B**) Schematic representation of mitochondrial respiratory complexes assessed. (**C**) Summary of sex-specific changes in mitochondrial enzyme activities induced by metformin, including NADH–CoQ oxidoreductase (Complex I), succinate–cytochrome c reductase (Complexes II–III), cytochrome c oxidase (Complex IV), and ATP synthase (Complex V). Directionality of change is shown relative to untreated *Atp7b*^−/−^ mice(↑ increase, ↓ decrease, — no change). Quantitative data are provided in Supplementary Fig. 3. (**D**) Citrate synthase activity measured in mitochondria-enriched liver fractions (*n* = 18), expressed as nmol·min^−1^·mg protein^−1^. Data were analyzed by ANOVA followed by Tukey’s HSD post hoc test. (**E**) Lipid vacuole area (nm²) quantified in liver sections. Least-squares means ± SE are shown for treatment groups separated by sex, based on a full factorial ANOVA model including sex, treatment, and sex × treatment interaction, followed by Tukey’s HSD post hoc testing. (**F**) Reactive oxygen species (ROS) quantified in mitochondria-enriched liver fractions (*n* = 18), expressed as log2 of the rate of hydrogen peroxide production expressed as nmol H_2_O_2_ produced x (min x mg protein)^−1^. Data were analyzed by ANOVA followed by Tukey’s HSD post hoc test.

The proportion of mitochondria within each classification was visualized using a contingency plot (Fig. 5A). At baseline, males and females exhibited similar distributions of fission- and fusion-associated morphology (females: fission = 5.1%, fusion = 4.5%, none = 90.4%; males: fission = 3.4%, fusion = 3.9%, none = 92.7%). Following metformin treatment, a sex-dependent response emerged. Females exhibited an approximately 5-fold increase in fusion-associated mitochondria, whereas males displayed only a ~ 2-fold increase (females: fission = 1.9%, fusion = 22.8%, none = 75.2%; males: fission = 2.6%, fusion = 8.6%, none = 88.7%). Thus, a significant sex × treatment effect was observed, with females showing a disproportionately greater shift toward fusion-associated morphology. In both sexes, the proportion of mitochondria exhibiting fission-associated features decreased by approximately 50% following metformin treatment.

In summary, at baseline, male and female *Atp7b*^−/−^ mice exhibited comparable distributions of mitochondria associated with fission and fusion. After metformin treatment, females demonstrated a pronounced increase in fusion-associated mitochondrial morphology, whereas males showed a more modest response. In both sexes, fission-associated features declined. Together, these findings indicate that metformin is associated with a greater shift toward fusion-like mitochondrial morphology in females, suggesting altered structural remodeling under copper-induced stress.

### Sex-dependent effects of metformin on mitochondrial bioenergetics and oxidative stress

Functional analyses revealed that divergent bioenergetic outcomes accompanied the observed structural remodeling. Activities of Complex I (NADH–CoQ oxidoreductase; NQR), Complexes II–III (succinate–cytochrome c reductase; SCCR), Complex IV (cytochrome c oxidase; CCO), Complex V (ATPase), and citrate synthase (CS) were measured in mitochondria-enriched fractions (Fig. 5B). Data were analyzed using least-squares fitting followed by ANOVA to assess overall model quality and interactions among sex, treatment, and their interaction (Table 1). The global model was significant, indicating that the selected variables robustly explained variance in enzyme activity. Several enzymatic activities were influenced by sex (Complex I, II–III, V, and citrate synthase), treatment (Complex II–III, IV, and citrate synthase), and sex × treatment interaction (Complex IV and citrate synthase) (Fig. 5C). Post hoc analysis using Tukey’s HSD test showed that, at baseline, citrate synthase activity was 22% higher in baseline males than in females (Fig. 5D).

**Table 1:** Analysis of mitochondrial bioenergetics: ANOVA *P* values for main effects (Sex, treatment) and their interaction Analysis of variance (two-way factorial ANOVA) was performed using the Fit Model platform in JMP 18 (SAS Institute), with sex and metformin treatment as fixed factors and their interaction (sex × treatment) included in the model. Reported *P* values correspond to F-tests for each main effect and interaction term.

Activity	Sex	Metformin treatment	Sex x treatment	*P*_Overall_
Complex I	**0.0081**	0.1642	0.1409	**0.0106**
Complex II-III	**0.0015**	**< 0.0001**	0.2165	**< 0.0001**
Complex IV	0.7160	**0.0004**	**0.0166**	**0.0010**
Complex V	0.0825	0.1965	0.2897	0.1338
Citrate synthase	0.0991	**0.0115**	**0.0120**	**0.0051**
Effect summary	**0.00018**	**< 0.0001**	**0.01202**	*–*

Sample sizes were as follows: untreated females (*n* = 4), untreated males (*n* = 4), metformin-treated males (*n* = 5), and metformin-treated females (*n* = 5). Statistical significance was assessed at α = 0.05, with significant values indicated in bold.

Following metformin treatment, mitochondria from females exhibited a 46% increase in Complex I activity relative to treated males (Supplementary Fig. 3A). Complex II–III activity increased markedly in both sexes, with a 2.8-fold increase in females and a 3.7-fold increase in males relative to their respective untreated *Atp7b*^−/−^ mice; however, treated females still exhibited significantly higher activity than treated males (1.6-fold difference; Supplementary Fig. 3B). In contrast, metformin-treated males displayed a 25% reduction in Complex IV activity compared with untreated males (Supplementary Fig. 3C), whereas females showed no change in Complex IV activity. No treatment- or sex-dependent changes were observed in Complex V activity (Supplementary Fig. 3D). Citrate synthase activity was additionally reduced by 19% in treated males compared with untreated males (Fig. 5D). Together, these findings demonstrate that mitochondrial enzyme activities are differentially regulated by sex in response to metformin in the WD model, with females showing enhanced electron transport capacity and males exhibiting reductions in key markers of mitochondrial content and function.

To assess downstream metabolic consequences, lipid vacuole morphometry was quantified. At baseline, lipid vacuole area and perimeter did not differ between sexes. Following metformin treatment, males exhibited larger lipid vacuoles than females, as reflected by increased vacuole area (*P* < 0.0001; Fig. 5E) and perimeter (*P* = 0.0109; Supplementary Fig. 3E). These findings indicate a sex difference in treatment-associated hepatic lipid vacuole morphology.

Because mitochondrial reactive oxygen species (ROS) production reflects inefficiencies in electron transport, ROS generation was evaluated as a marker of mitochondrial dysfunction and oxidative stress. ROS levels showed a significant interaction with sex (*P* < 0.0001; Fig. 5F), with higher ROS production in metformin-treated males than in treated females. Significant sex × treatment interactions were also detected (*P* = 0.0002 and *P* < 0.0001), with both treated and untreated females exhibiting lower ROS production than males. Importantly, ROS levels were comparable between sexes in the untreated groups, indicating that sex-dependent differences in oxidative stress emerged primarily in response to metformin.

In summary, metformin enhanced mitochondrial electron transport capacity in females, as evidenced by increased Complex I and II–III activities. In contrast, males exhibited reductions in Complex IV and citrate synthase activities, indicating diminished mitochondrial oxidative capacity. These bioenergetic differences were accompanied by downstream metabolic consequences: metformin-treated males accumulated larger hepatic lipid vacuoles—consistent with impaired fatty acid β-oxidation—and produced higher levels of mitochondrial ROS than females. Notably, baseline oxidative stress was similar between sexes, underscoring that the observed divergence arises specifically in response to metformin treatment rather than from preexisting sex differences.

### Metformin differentially modulates hepatic stress hormones and lipid mediators

Based on evidence that glucocorticoids (e.g., dexamethasone) modulate mitochondrial function through regulation of the fission protein Drp1^42^ and influence mitochondrial fusion–fission balance, we hypothesized that hepatic copper accumulation in WD initiates a stress response that alters hormone signaling and lipid metabolism. This framework provides a physiological context for linking mitochondrial remodeling with systemic endocrine responses. Under stress, corticotropin-releasing hormone (CRH) released from the hypothalamus stimulates pituitary secretion of adrenocorticotropic hormone (ACTH), which in turn drives adrenal glucocorticoid release. Chronic stress, as observed in WD, may preferentially affect the hypothalamic–pituitary–gonadal (HPG) axis through activation of the hypothalamic–pituitary–adrenal (HPA) axis. Dysregulation of these interconnected axes has been shown to influence mitochondrial function, oxylipin balance, and reproductive hormone signaling^43–46^. Accordingly, we evaluated hepatic glucocorticoids, oxylipins, and sex hormones to determine whether metformin modifies stress-related metabolic pathways in a sex-dependent manner.

**Fig. 6:**
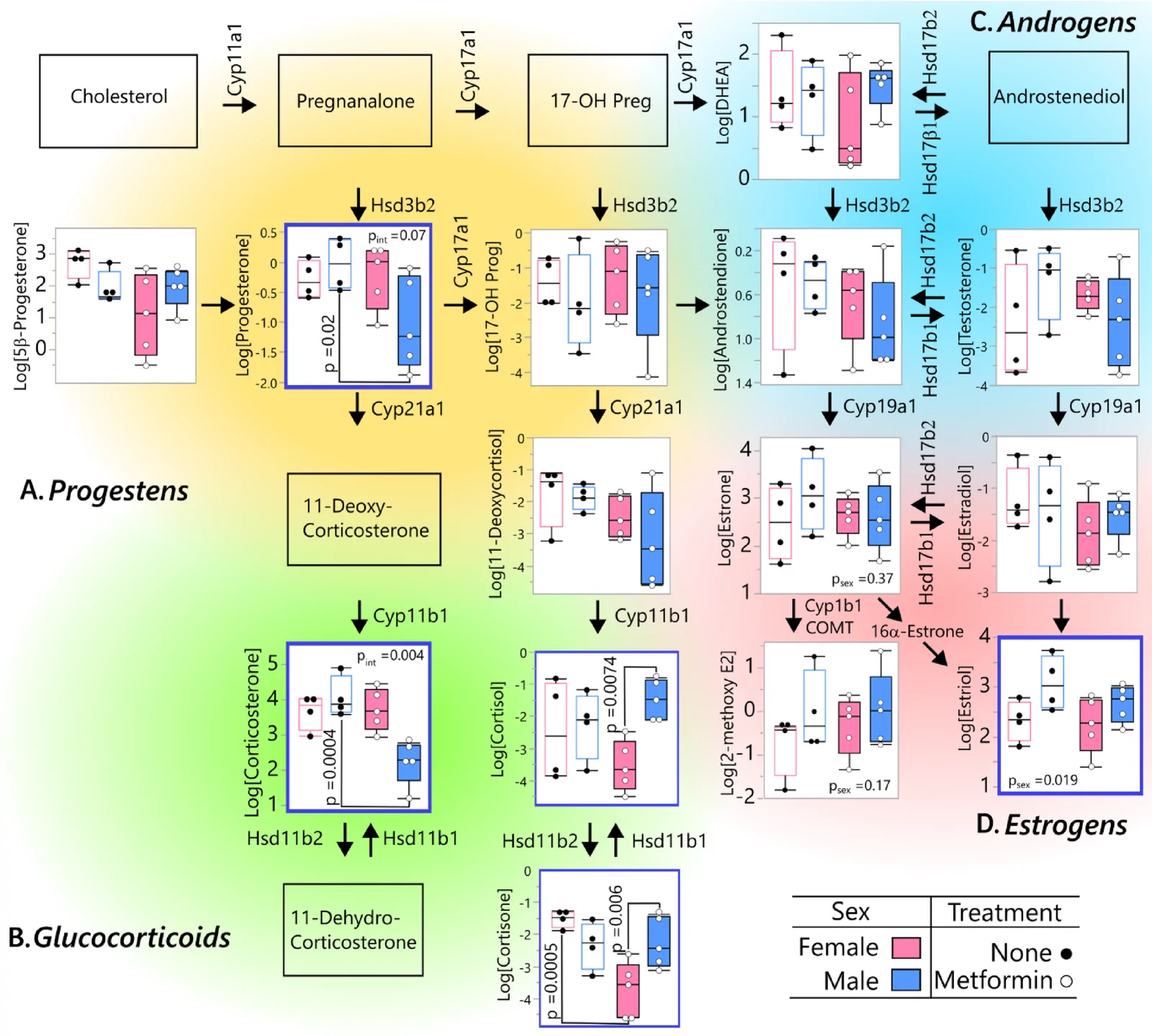
Sex- and treatment-dependent modulation of hepatic steroid hormone pathways in Atp7b^−/−^ mice. Schematic representation of hepatic steroid hormone biosynthetic and metabolic pathways analyzed in female and male *Atp7b*^−/−^ mice. Hormone classes are color-coded as follows: progestens (yellow), glucocorticoids (green), androgens (blue), and estrogens (red). Enzymatic reactions are indicated between compounds, and metabolites not directly visualized are shown as boxed intermediates. Statistical annotations indicate significant effects of sex, treatment, or the sex × treatment interaction, derived from a mixed-effects ANOVA with least-squares means contrast post hoc testing. Compounds outlined with blue boxes denote statistically significant differences (*P* < 0.05).

We quantified primary sex hormones (estradiol, progesterone, testosterone, estriol, and estrone), precursor and intermediate hormones (DHEA, androstenedione, and 17-hydroxyprogesterone), and metabolites of estrogen and progesterone (5β-hydroxyprogesterone and 2-methoxyestradiol). Among primary sex hormones, estriol and estrone exhibited significant sex interactions (*P* = 0.019 and *P* = 0.037, respectively; Fig. 6A). Progesterone showed a treatment effect in males (*P* = 0.02).

Within the glucocorticoid family, we quantified cortisol, cortisone (the inactive metabolite of cortisol generated by 11β-HSD2), corticosterone, and 11-deoxycortisol. Corticosterone is the primary glucocorticoid in rodents^47^; however, recent studies indicate that both cortisol and corticosterone are present in rodent circulation and respond to distinct metabolic and physiological stressors, with cortisol reflecting acute stress and corticosterone more closely associated with chronic stress^45^. At baseline, males and females exhibited comparable hepatic levels of cortisol, corticosterone, and cortisone. Following metformin treatment, females exhibited significantly lower cortisol levels compared with untreated females and treated males, indicating a significant sex × treatment interaction (*P* = 0.0074; Fig. 6B). Corticosterone levels were significantly reduced in treated males (*P* = 0.0004), whereas cortisone levels were reduced in treated females relative to untreated females (*P* = 0.0005) and treated males (*P* = 0.006). Thus, metformin treatment was associated with sex-specific differences in hepatic glucocorticoid profiles, producing divergent hormonal responses despite similar baseline levels. Levels of 11-deoxycortisol were unaffected by sex or treatment. Collectively, these findings indicate sex-dependent modulation of HPA-axis–related glucocorticoids in the WD liver, with metformin exerting distinct regulatory effects in males and females.

No significant differences were observed among hormone precursors, intermediates, or estrogen and progesterone metabolites (Fig. 6C,D). Overall, sex hormone alterations were limited and selective, indicating that metformin does not broadly disrupt hepatic sex steroid metabolism in this model.

Oxylipin profiling revealed additional sex-dependent effects (Supplementary Fig. 4). At baseline, untreated females exhibited higher levels of lipoxygenase- and cyclooxygenase-derived oxylipins compared with untreated males. Metformin treatment was associated with a marked reduction in these oxylipins in females, whereas in males, there were minimal changes. These findings suggest that inflammatory lipid mediator pathways are more active at baseline in females and more responsive to metformin treatment.

In summary, baseline hepatic glucocorticoid levels were comparable between sexes. Metformin reduced cortisol and cortisone predominantly in females, whereas corticosterone was reduced primarily in males, resulting in sex-divergent glucocorticoid profiles. Sex hormone analysis revealed modest and selective effects, limited to estriol, estrone, and progesterone. Oxylipin profiling demonstrated higher baseline inflammatory lipid mediators in females that were selectively attenuated by metformin, while males exhibited minimal oxylipin modulation (Supplementary Fig. 4). Together, these data indicate that metformin differentially modulates hepatic stress hormone signaling and lipid mediator pathways in a sex-dependent manner, potentially contributing to the divergent mitochondrial and metabolic responses observed in *Atp7b*^−/−^ mice relative to wild-type^11–15^.

### Metformin reduces FXR antagonism and alters bile acid conjugation in females

**Fig. 7:**
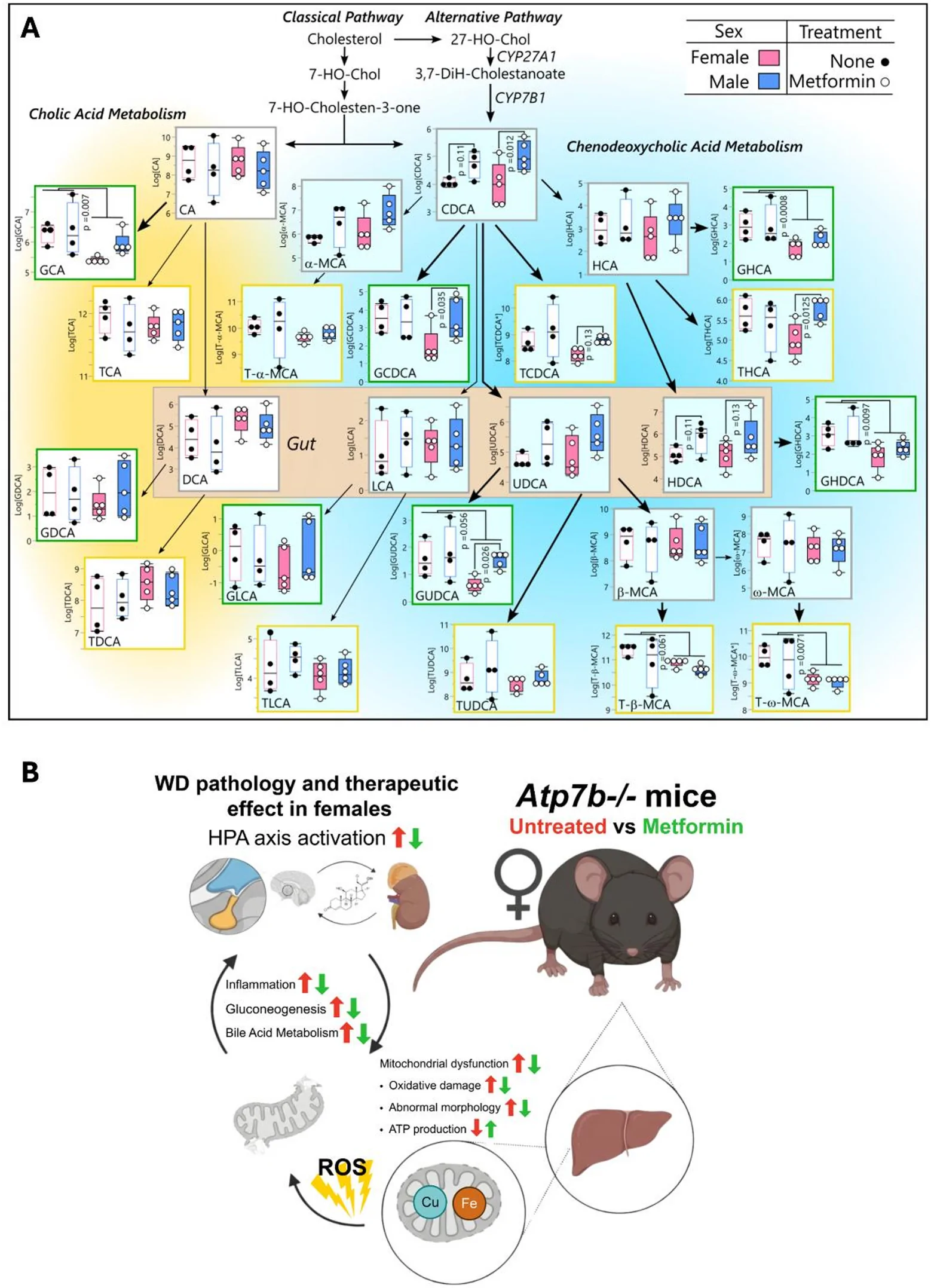
Sex-dependent modulation of bile acid metabolism and stress-related pathways by metformin in Atp7b^−/−^ mice. (**A**) Schematic overview of classical and alternative bile acid metabolic pathways in female and male *Atp7b*^−/−^ mice, highlighting sex- and treatment-dependent effects. Cholic acid (CA)–derived pathways are shown on a gold background, and chenodeoxycholic acid (CDCA)–derived pathways on a blue background. Individual bile acids are outlined by conjugation status: unconjugated (gray), glycine-conjugated (green), and taurine-conjugated (gold). Secondary bile acids generated by the gut microbiome are enclosed within the brown box. Sex-specific differences are indicated by pink (female) and blue (male) annotations, and dot shading denotes treatment effects. Displayed P values denote statistically significant effects or trends, as described in the Results. (**B**) Integrated model summarizing sex-specific effects of metformin on Wilson disease–associated hepatic pathology in *Atp7b*^−/−^ mice. In untreated conditions, hepatic mitochondria exhibit abnormal morphology, copper and iron accumulation, elevated oxidative stress, and activation of stress-related hormonal signaling associated with hypothalamic–pituitary–adrenal (HPA) axis output. Metformin treatment is associated with improved mitochondrial structure and function, reduced oxidative stress, enhanced ATP production, and attenuation of stress-related hormonal signaling and bile acid dysregulation. These coordinated effects are most pronounced in females.

Tauro-β-muricholic acid (T-β-MCA) was the dominant conjugated bile acid in *Atp7b*^−/−^ mice, accounting for approximately 27% of the total conjugated bile acid pool and nearly 60% of taurine-conjugated muricholic acids. Metformin treatment reduced hepatic T-β-MCA levels in both sexes, with a trend toward significance (*P* = 0.061; Fig. 7A). Because T-β-MCA has been reported to function as an antagonist of the farnesoid X receptor (FXR), its reduction would be expected to alter the relative abundance of bile acids with reported FXR antagonistic versus agonistic activity. To approximate this balance, we calculated a ratio of T-β-MCA to the sum of bile acids reported to act as FXR agonists (chenodeoxycholic acid [CDCA], deoxycholic acid [DCA], lithocholic acid [LCA], and their taurine conjugates^48–50^. At baseline, this ratio was approximately 2.5-fold higher in females than in males. Following metformin treatment, the ratio decreased by approximately 50% in females but was not significantly altered in males (Fig. 7A). These findings indicate that metformin shifted the composition of the bile acid pool in females toward a lower relative abundance of the FXR antagonist T-β-MCA compared with bile acids reported to activate FXR.

Metformin also altered bile acid conjugation patterns, as reflected by an increase in the taurine-to-glycine conjugate ratio. This effect was driven entirely by a reduction in primary glycine-conjugated bile acids in females, including glycocholic acid (GCA; *P* = 0.007), glycohyocholic acid (GHCA; *P* < 0.001), glycoursodeoxycholic acid (GUDCA; *P* = 0.056), and glycohyodeoxycholic acid (GHDCA; *P* < 0.001). In contrast, levels of primary and secondary taurine-conjugated bile acids were unchanged. The summed pools of unconjugated primary, unconjugated secondary, conjugated primary, and conjugated secondary bile acids showed no significant differences by sex, treatment, or sex × treatment interaction (Fig. 7A). Similarly, primary-to-secondary bile acid ratios were unchanged, arguing against a major metformin-induced alteration in microbial 7α-dehydroxylation activity (Fig. 7A). The preservation of unconjugated bile acid pools, together with selective depletion of glycine conjugates, supports altered hepatic conjugation rather than enhanced microbial deconjugation.

In sum, tauro-β-muricholic acid—a dominant bile acid and FXR antagonist^50^—was elevated in *Atp7b*^−/−^ mice and modestly reduced by metformin relative to pre-established wildtype controls^11–15^. Females exhibited greater baseline FXR antagonism, which was substantially attenuated by metformin treatment, whereas males showed no significant change. Metformin selectively reduced glycine-conjugated bile acids in females without affecting total bile acid pool size or primary-to-secondary bile acid ratios, indicating a sex-specific, liver-intrinsic effect on bile acid conjugation and FXR signaling rather than a microbiome-driven mechanism.

### Integrated summary of results

Collectively, these data demonstrate that female *Atp7b*^−/−^ mice, despite exhibiting greater baseline mitochondrial injury and hepatic metal burden, exhibit a coordinated set of structural, biochemical, and hormonal changes in response to metformin. This response is characterized by enhanced mitochondrial fusion, structural remodeling, increased respiratory complex activity, reduced oxidative stress, and attenuation of hepatic stress hormone signaling (Fig. 7B). In contrast, male mice display comparatively limited mitochondrial plasticity and exhibit unfavorable bioenergetic and oxidative outcomes despite equivalent metformin exposure. Together, these findings establish sex as a critical determinant of mitochondrial adaptation and metabolic response to metformin in the setting of WD.

## Discussion

WD is an inherited disorder of copper metabolism characterized by progressive copper accumulation in the liver, brain, and other organs, leading to mitochondrial toxicity and systemic metabolic dysfunction. Mitochondria are particularly vulnerable to copper overload because they serve as major intracellular sites for copper handling and redox reactions. Prior work, including our own, has demonstrated that WD is associated with profound mitochondrial abnormalities, including mtDNA depletion, impaired ATP production, increased ROS, and severe ultrastructural disruption^11,13,18,39,51,52^. These mitochondrial defects are increasingly recognized as contributors to hepatocellular dysfunction and to the systemic metabolic and neuropsychiatric manifestations of WD^53,54^.

Metformin is a widely prescribed antihyperglycemic agent with well-established effects on mitochondrial metabolism, cellular redox state, and stress signaling. Although its glucose-lowering efficacy is generally comparable between men and women^55,56^, a growing body of literature documents sex-dependent—and in some cases opposing—biological effects of metformin across diverse systems, including longevity, tumorigenesis, endocrine regulation, neurological recovery, musculoskeletal outcomes, and metabolic disease [4, 5,^57^]. These observations highlight the importance of evaluating sex as a biological variable when investigating metabolic therapies in non-diabetic disease contexts such as WD.

In this study, we investigated whether metformin could restore mitochondrial structure and function in a murine model of WD and whether therapeutic responses differed by sex. Two principal observations emerge from this study. First, *Atp7b*^−/−^ mice accumulate excess copper and iron within hepatic mitochondria, resulting in marked mitochondrial dysfunction and a chronic cellular stress state that engages endocrine and metabolic pathways. Second, metformin is associated with sex-specific mitochondrial responses, characterized by broader structural and biochemical changes in females and more limited or divergent changes in males. Together, these findings establish sex as a major determinant of mitochondrial pathology and therapeutic response in WD^3^.

At baseline, female *Atp7b*^−/−^ mice exhibited higher hepatic and mitochondrial copper and iron levels than males, suggesting that mitochondria may represent a compartment in which metal accumulation is prominent in WD. This phenomenon appears to be unique to WD and reflects a disease-specific disruption of iron–copper homeostasis^58^. Correspondingly, females displayed more pronounced mitochondrial ultrastructural abnormalities. Notably, we identified mitochondrial protrusions giving rise to small, electron-dense mitochondria-derived vesicles (MDVs)—a finding not previously reported in WD. MDVs are increasingly recognized as components of mitochondrial quality-control pathways, enabling selective removal of damaged proteins, lipids, or metals without complete mitochondrial turnover. In the context of WD, the enrichment of electron-opaque material within MDVs raises the possibility of a mechanism involved in handling damaged or metal-associated mitochondrial components, although its functional significance was not directly assessed.

Quantitative morphometric analyses further revealed that female mitochondria were smaller at baseline and exhibited higher perimeter-to-area and surface-to-volume ratios, consistent with increased fragmentation or structural stress. These features have been associated with mitochondrial injury, an altered fusion-fission balance, and mitophagy susceptibility in other contexts. Importantly, overall shape parameters were preserved, indicating constrained but not catastrophic remodeling. Together, these baseline features indicate that female mitochondria exhibit greater ultrastructural alteration while maintaining preserved shape parameters.

Sex-specific differences extended beyond mitochondrial morphology to endocrine and metabolic profiles. Males showed higher baseline levels of hepatic estrogen metabolites, which were not directly examined in this study but may indicate compensatory responses to copper-induced disruption of steroid metabolism. Hepatic glucocorticoid metabolism is also strongly sex-dependent, shaped in part by differential expression of 11β-hydroxysteroid dehydrogenase enzymes^59^. These intrinsic differences likely contribute to divergent mitochondrial resilience, as female liver mitochondria exhibit more efficient NADH-linked respiration and generate less hydrogen peroxide than male mitochondria^60^. Enhancement of NADPH availability preferentially strengthens antioxidant defenses and suppresses inflammatory responses in females^61^, providing a potential biochemical context for sex-associated differences in mitochondrial responses.

Importantly, metformin treatment did not significantly reduce hepatic or mitochondrial copper levels as measured by ICP-MS, in agreement with prior studies across multiple disease models^26,27,62^. Because copper accumulation is the primary pathogenic driver in WD, these findings indicate that the observed improvements in mitochondrial morphology and respiratory function, particularly in females, are unlikely to result from reduced copper burden. Rather, they suggest that metformin may modulate mitochondrial stress responses or metabolic demand independently of copper removal, as observed elsewhere^63–65^. For example, metformin suppresses hepatic gluconeogenesis and alters cellular energy balance, potentially reducing mitochondrial metabolic demand under conditions of copper-induced stress. Such metabolic unloading may allow mitochondria to shift toward fusion-associated morphology and improved structural organization despite persistent copper accumulation. Thus, the mitochondrial improvements observed here likely reflect metabolic and bioenergetic modulation rather than direct correction of copper overload.

Structural remodeling in response to metformin was markedly sex dependent. In females, mitochondria became larger, rounder, and structurally smoother, with increased circularity and solidity and reduced perimeter-to-area and surface-to-volume ratios. These features are consistent with a shift toward more fused and structurally regular mitochondrial networks, although fusion–fission dynamics were not directly measured. UMAP analysis revealed clear segregation of female mitochondria following treatment, whereas male mitochondria largely overlapped across conditions. These structural changes were accompanied by differences in mitochondrial enzymatic or carrier activities and reduced ROS production in females. In contrast, male *Atp7b*^−/−^ mice exhibited limited mitochondrial plasticity. Although Complex II–III activity increased modestly, males showed reductions in Complex IV and citrate synthase activity, increased lipid vacuolization, and elevated mitochondrial ROS. Morphometric features were consistent with less coordinated structural remodeling compared to females. While females achieved approximately 1.2-fold higher circulating metformin levels—consistent with known sex differences in absorption and clearance^66^—these concentrations remained within clinically relevant ranges^65,67,68^, suggesting that differences in metformin exposure alone are unlikely to fully account for the observed sex-dependent responses. Thus, these findings indicate a distinct pattern of mitochondrial response to metformin in males, unlikely to be explained solely by differences in drug exposure.

Intrinsic sex differences in mitochondrial redox capacity and lipid signaling may contribute to the observed differences in structural responses^69^. Metformin is known to influence cellular redox state through shifts in NADH/NAD⁺ and NADPH/NADP⁺ ratios^67^, which could interact with pre-existing sex-dependent differences in antioxidant capacity. Such interactions may provide a biochemical context for the more pronounced mitochondrial remodeling observed in females, although these mechanisms were not directly assessed in the present study.

Metformin was also associated with sex-specific changes in endocrine and bile acid signaling. In females, hepatic cortisol and cortisone levels were reduced, whereas corticosterone was preferentially reduced in males. These differences may reflect known sex-dependent regulation of the hypothalamic–pituitary–adrenal (HPA) axis^70^ and could be related to differences in mitochondrial stress responses, although causal relationships were not directly tested.

Metformin treatment was associated with a reduction in tauro-β-muricholic acid (T-β-MCA), a dominant bile acid and FXR antagonist, particularly in females. This change may reduce FXR antagonism without altering total bile acid pool size or primary-to-secondary bile acid ratios, suggesting a liver-intrinsic effect on bile acid metabolism rather than microbiome-driven changes. Given prior links between FXR signaling, fatty acid metabolism, and mitochondrial function^71^, these observations raise the possibility that bile acid remodeling contributes to mitochondrial adaptations; however, this relationship remains correlative and requires direct mechanistic investigation.

Collectively, our findings demonstrate that sex is an important determinant of mitochondrial pathology and response to metformin in WD. Despite greater baseline mitochondrial injury and hepatic metal burden, female *Atp7b*^−/−^ mice exhibited more pronounced structural remodeling and broader treatment-associated changes, including alterations in mitochondrial morphology, respiratory parameters, oxidative stress markers, and endocrine and bile acid profiles. In contrast, male mice showed fewer structural and bioenergetic changes. These findings support a model in which metformin induces sex-dependent mitochondrial adaptations rather than uniform improvement, and suggest that sex-specific mitochondrial biology should be considered in the design and interpretation of therapeutic strategies for WD.

### Study limitations

Several limitations should be considered when interpreting these findings. First, this study was conducted exclusively in a murine model of WD, and the extent to which these observed sex-dependent mitochondrial responses translate to human patients remains unknown. Second, the number of animals per group was relatively small (total *n* = 18), which may limit statistical power and the generalizability of the findings; therefore, the results should be interpreted with appropriate caution. However, this limitation was partially mitigated by the large number of mitochondria analyzed across transmission electron microscopy images. Third, metformin exposure was relatively short (2 weeks), and longer-term effects on mitochondrial turnover, liver function, and disease progression were not evaluated. Fourth, while extensive structural, biochemical, and hormonal analyses were performed, we did not directly measure mitochondrial biogenesis, mitophagy flux, apoptosis, or in vivo hepatic functional outcomes such as serum transaminases or bile flow. Fifth, mitochondrial dynamics were inferred from morphometric features rather than direct measurement of fission–fusion regulatory proteins (e.g., DRP1, MFN1/2, OPA1) or live-cell imaging. Therefore, the observed changes should be interpreted as morphological associations consistent with altered mitochondrial dynamics, rather than definitive mechanistic evidence of fission–fusion regulation. In addition, metformin treatment did not significantly reduce hepatic or mitochondrial copper levels. Therefore, the observed mitochondrial improvements should not be interpreted as evidence of copper removal but rather as metabolic effects that may partially mitigate mitochondrial stress under copper overload. Finally, although sex-dependent differences in metformin exposure were observed, the study was not designed to dissect the relative contributions of drug exposure versus intrinsic sex-dependent mitochondrial differences. Taken together, these limitations indicate that the present findings are hypothesis-generating and warrant further mechanistic and translational investigation.

### Translational and clinical implications

Collectively, our findings demonstrate that sex is a critical biological determinant of mitochondrial dysfunction and therapeutic response in WD. Despite greater baseline mitochondrial injury and hepatic metal burden, female *Atp7b*^−/−^ mice exhibit significant mitochondrial plasticity and show broader treatment-related changes after metformin, including improved mitochondrial dynamics, enhanced respiratory function, reduced oxidative stress, and beneficial modulation of endocrine and bile acid signaling. In contrast, male mice display limited mitochondrial adaptability and less favorable bioenergetic outcomes. These results suggest that metformin may warrant further investigation as a sex-informed adjunctive approach to copper chelation therapies, particularly in female patients with WD who exhibit prominent metabolic or stress-related features. It is also important to note that our work provides a scoring system for assessment and quantification of mitochondria TEM morphology, which could be used in models of WD and beyond. More broadly, this work underscores the need to incorporate sex as a stratifying variable in both preclinical and clinical trials of metabolic therapies for WD and supports a framework that considers sex-specific mitochondrial biology in the design and interpretation of future therapeutic studies.

## Methods

### Animal model and experimental design

*Atp7b*^−/−^ global knockout mice on a C57BL6 background, provided by Dr. Svetlana Lutsenko (Johns Hopkins University), were bred in-house on the UC Davis campus. Mice were group-housed in polycarbonate cages and kept at 20–23 °C with 45–65% relative humidity, following a 14-hour light/10-hour dark cycle. The UC Davis Institutional Animal Care and Use Committee approved all husbandry and experimental procedures, adhering to the National Research Council’s Guide for the Care and Use of Laboratory Animals. The *Atp7b*^−/−^ mice (12–14 weeks old) were given free access to food and water. They were randomly assigned to receive either standard vivarium deionized drinking water (*n* = 8, 4 M/4F) or metformin-supplemented water (500 mg/kg/day; *n* = 10; 5 M/5F) for two weeks using a computer-generated randomization scheme. The metformin dose was based on average body weight and water intake; body weight was recorded on days 1, 4, 8, and 14, while water and food intake were measured on days 1, 5, 9, and 15. The metformin concentration in water was adjusted as needed to maintain a steady dose. At study completion, fed mice were anesthetized with isoflurane and euthanized by retro-orbital exsanguination followed by cervical dislocation. Mice were euthanized between 9–11am. Blood was collected in K_2_EDTA tubes and centrifuged at 6000 x g for 10 min. Plasma was aliquoted and stored at − 80 °C. Livers were collected for downstream analyses with portions placed in ice-cold PBS, flash-frozen in liquid nitrogen, or placed in electron microscopy fixative. Frozen livers were stored at − 80 °C until further analysis. Liver portions in ice-cold PBS were processed for mitochondria isolation the same day after dissection. Livers for electron microscopy were processed and imaged within the same week of collection.

### Plasma metformin quantification

Plasma metformin levels were measured using ultra-performance liquid chromatography tandem mass spectrometry (UPLC-MS/MS) after protein precipitation in a 96-well plate format. Briefly, 10 µl of plasma was spiked with 10 µl of 50 µM d6-metformin in acetonitrile, along with 10 µl of a 1 µM solution of CUDA/PUHA in acetonitrile, followed by the addition of 70 µl of chilled acetonitrile containing 0.1% acetic acid. The samples were vortexed for 1 min, centrifuged at 2247 g and 4 °C for 15 min, and the supernatant was filtered through a 0.2 μm PVDF filter plate (Agilent Technologies, Santa Clara, CA) for 2 min under the same centrifugation conditions. The extracts were analyzed on an Acquity UPLC (Waters Corp, Milford, MA) and separated using a 2 × 150 mm, 3 μm Luna Silica (2) column (Phenomenex, Torrance, CA) maintained at 35 °C, with gradient elution at a flow rate of 0.4 ml/min. Solvent A consisted of water with 0.1% acetic acid, and solvent B was acetonitrile with 0.1% acetic acid. The gradient was set as follows: 2% B from 0 to 0.5 min, increased to 13% B from 0.5 to 2.5 min, then to 100% B from 2.5 to 3 min. B was maintained at 100% from 3 to 4.5 min, after which the column was reverted to initial conditions over 0.1 min, and held at 2% B from 4.6 to 6.5 min. Analytes were detected by positive-mode electrospray ionization using multiple-reaction monitoring on a 4000 QTRAP (Sciex; Redwood City, CA).

### Tissue collection and mitochondrial isolation

Following euthanasia, liver tissue was excised and subdivided for ultrastructural, biochemical, and molecular analyses. Mitochondria were isolated using the Mitochondria Isolation Kit for Tissue (Pierce Biotechnology, catalog #89801, Rockford, IL, USA). Reagents were prepared according to the kit manual to achieve the required volume for the aliquots. Reagent A was prepared with enough BSA (provided in the kit) to create a 4 mg/mL solution. Reagent C was placed in a separate tube. Before use, one tablet of Complete™ Mini EDTA-free Protease Inhibitor Cocktail (Roche #11836170001, Mannheim, Germany) was dissolved in Reagent A along with bovine serum albumin and in the Reagent C tubes. We followed protocol #2 of Option A (Dounce homogenization of soft tissues), as described in the kit manual. After dissection, approximately 600 mg of exsanguinated liver from each mouse (*n* = 18) was kept in ice-cold PBS. After draining the excess of phosphate buffer saline (PBS), samples were rapidly minced with surgical scissors, and 2400 µl of reagent A was added to each. Dounce grinding was immediately performed using a hand-held PRO Scientific Bio-Gen PRO200 homogenizer (Cole-Parmer, Vernon Hills, IL) attached to a motorized unit (Glas-Col Continuous Duty D.C. Motor, Terre Haute, IN, USA) so that the Teflon pestle rotated at 25 rpm. The glass dounce cylinder holding the sample was manually raised against the rotating pestle for three strokes or until no solid tissue was visible. Each homogenized sample was divided into three equal aliquots in 2 mL centrifuge tubes, and 800 µl of mitochondrial isolation reagent C was added to each. The tubes were inverted to mix. Aliquots were centrifuged at 700 g for 10 min at 4 °C. The resulting pellet (cellular debris) was discarded, and the supernatant was transferred to another 2 mL centrifuge tube. These tubes were centrifuged at 3000 g for 15 min at 4 °C. The supernatant (cytosolic fraction) was removed and stored separately for analysis. The remaining mitochondrial pellet was washed with PBS and centrifuged at 12,000 g for 5 min. The supernatant was discarded. The mitochondrial pellet and cytosolic fractions were stored at − 80 °C until further analysis.

### Transmission electron microscopy (TEM) imaging

TEM imaging of mouse livers began by fixing liver samples with glutaraldehyde to preserve cellular structures via protein cross-linking. This was followed by a secondary fixation in osmium tetroxide to stabilize lipids and improve contrast. The tissues were then dehydrated through a graded ethanol series and embedded in epoxy resin. Ultra-thin sections, typically about 50–70 nm thick, were cut with an ultramicrotome and mounted on copper grids. These sections were stained with uranyl acetate to increase electron contrast. Finally, the prepared grids were examined under a transmission electron microscope (TEM) to obtain detailed images of the liver’s ultrastructure, including organelles such as mitochondria and the endoplasmic reticulum. The sample processing and TEM imaging were performed at the UC Davis Tupper Hall Tissue/Cellular EM Facility for a service fee.

### Quantitative analysis of mitochondrial ultrastructure

Quantification analysis methods were developed using the National Institutes of Health (NIH) ImageJ software. ImageJ is an open-source image processing program that analyzes multidimensional scientific images, including TEM and confocal microscopy datasets. The pool of analyzed images (*n* = 196) included 40 images from the female untreated group, 44 from the male untreated group, 50 from the female metformin group, and 60 from the male metformin group (Supplementary Table 1). All images had a resolution of 4096 × 4096 pixels. Most images had a resolution of approximately 0.933 pixels/nm (*n* = 185), except for images from mice, which had a resolution of 0.733 pixels/nm (five untreated and four treated males). Each image contained a scale bar, the length of the bar, and the magnification required to set the appropriate units in ImageJ. Mitochondrial parameters were based on our previous studies^41,72^ and summarized in Supplementary Table 3.

To ensure an unbiased approach, mitochondrial outcomes were quantified in a randomized and blinded manner. Independent reviewers conducted the image analysis to minimize bias in the results. Quantification was performed separately by two reviewers per image, in a randomized, blinded process. In the measurement phase, seven reviewers analyzed the TEM images in this study and then cross-checked each other’s measurements. This process ensured rigorous evaluation and reduced the likelihood that individual biases would affect the outcomes. Additionally, any significant measurement discrepancies between reviewers were automatically flagged when the coefficient of variation (CV%) exceeded 20%, prompting further review and ensuring data reliability. By involving multiple independent reviewers and implementing cross-checking, the study effectively reduces potential bias and enhances the credibility of the image analysis outcomes. The following section will thoroughly explain the methods for assigning images, conducting analysis, and reviewing results. Images were stratified into folders containing 9–20 images per mouse. These folders were randomly assigned, and each reviewer analyzed 20–105 images from 2 to 9 mice across measurement rounds. Initial reviewers identified, circled, and numbered all non-cropped mitochondria in each image using Notability (Version 14.8) or Microsoft Paint (Windows 11), saving labeled reference images for subsequent quantitative measurements. The original images were then uploaded to ImageJ (Version 1.54) for measurement. The scale was set by manually measuring the scale bar length with the ‘Straight’ tool to generate a straight line segment. ‘Analyze’ > ‘Set Scale’ displays the pixel length of the line segment, along with the known distance, pixel aspect ratio, and unit of length. The known distance was entered as the scale bar length in nanometers (nm). The pixel aspect ratio was set to 1.0, and the unit was set as ‘nm.’ Parameters were set in ImageJ via ‘Analyze’ > ‘Set Measurements,’ selecting ‘Area,’ ‘Shape descriptors,’ ‘Mean grey value,’ ‘Perimeter,’ and ‘Fit ellipse.’ Each mitochondrion in the TEM images was traced along the outer perimeter of the outer membrane using the cursor and freehand selection tool. Mitochondria were measured with the parameters indicated on the labeled reference images. Area (nm²), mean pixel value, perimeter, major axis, minor axis, and solidity were recorded to three decimal places. Data were then imported into Microsoft Excel (Version 16.0). Measurements from ImageJ were pasted into an Excel template that automatically calculated the perimeter-to-area ratio (nm⁻¹), aspect ratio, integrated density (px²), and convex hull area (nm²). Additional parameters were calculated after the measurement phase. Once initial measurements were complete, folders were randomly reassigned to different reviewers for cross-checking, with two independent reviewers analyzing each folder. Cross-checkers received all images and labeled reference images to guide measurement order. They gathered the same parameters, and data were pasted into the Excel template alongside initial results. The template automatically calculated the average, standard deviation, and CV% for each parameter between initial and cross-checked measurements. CV% values of 20 or higher were automatically flagged. In such cases, cross-checkers re-measured mitochondria using labeled reference images to resolve discrepancies. All discrepancies were eventually resolved; no mitochondria were excluded from the analysis. Labeling, initial measurement, and cross-checking took approximately 5, 15, and 15 min per image per reviewer, respectively. Resolving CV% discrepancies typically required only one remeasurement, taking about two minutes per flagged mitochondrion.

### Mitochondrial injury scoring

Liver mitochondria were assessed based on four criteria designed to evaluate the overall organelle shape and the integrity of the outer and inner membranes, including cristae and the matrix. The mitochondrial shape was classified as spheroid, prolate, or oblong (score = 0), or circular or fragmented (score = 1). The major and minor axes were measured to aid in shape assessment and distinguish differences between shapes. A circular shape has approximately equal axes, while a spheroid, whether prolate or oblate, exhibits significantly different axis lengths. The outer membrane’s integrity was evaluated as intact and continuous, clearly separated from the inner membrane (score = 0), or mainly discontinuous or absent (score = 1). The inner membrane and cristae integrity were categorized as sharply defined, with less than 50% of the mitochondrial area devoid of cristae, lamellar, uniform in size, and symmetrically packed (score = 0), or mostly irregular, absent (> 50% of the area without cristae), fragmented, or dilated (score = 1). The overall mitochondrial score was derived by summing the scores for these criteria. Threshold values used to classify mitochondria as high risk for fission (perimeter/area > 0.01348) or fusion (solidity > 0.9905) were derived from nonparametric tolerance interval analysis and applied uniformly across groups.

### Evaluation of opaque or electron-dense mitochondrial deposits

To determine the number of electron-dense deposits, we needed to establish a cutoff diameter for inclusions to ensure consistent counting among reviewers. This was crucial for maintaining uniformity and minimizing the risk of mistakenly counting small subcellular structures, such as mitochondrial ribosomes, which are typically larger than cytosolic ribosomes and have an average diameter of about 30 nm. The threshold also helped differentiate these smaller structures from mitochondrial calcium deposits, which generally range from 50 to 500 nm and are often linked to pathological conditions. By defining a cutoff, we aimed to enhance the accuracy and reliability of the analysis, ensuring that only deposits meeting the specified criteria were included in the count. To achieve this, we measured the diameters of electron-dense inclusions in ImageJ in a stratified, representative subset of images and determined a standardized cutoff diameter (≥ 17 nm) for intramitochondrial electron-dense deposits. Seven reviewers counted the deposits from 1136 mitochondria following the protocol described for mitochondria scoring.

### Hepatic and mitochondrial metal quantification

Copper, iron, and zinc levels in liver tissue and isolated mitochondria were quantified by inductively coupled plasma mass spectrometry (ICP-MS). Samples were digested in nitric acid and hydrogen peroxide, diluted to volume, and analyzed using a Thermo iCapQ ICP-MS system with internal standards and multi-point calibration curves. Results are reported as µg metal per mg wet liver weight and as total ng per mitochondrial mass. Mouse liver samples (70–80 mg) and isolated liver mitochondria were digested at 65 °C in a matrix of 4 HNO3 (Fisher Scientific A509P500) and 1 H2O2 (Fisher Scientific NC1199178), then diluted with Milli-Q water to a total volume of 10 mL. A 10% HNO3 (v/v) solution served as the calibration blank. ICP-MS analysis was conducted using a computer-controlled (QTEGRA software) Thermo iCapQ ICP-MS (Thermo Fisher Scientific, Waltham, MA), equipped with an SC-2DX PrepFAST autosampler (Elemental Scientific Inc., Omaha, NE). An internal standard (Inorganic Ventures IV-ICPMS-71D, Christiansburg, VA) was added inline, and the PrepFAST system generated two calibration curves: 2000, 1000, 400, 200, 40, 20 ppb, and 100, 50, 20, 10, 2, and 1 ppb Cu. Samples underwent a 10x autodilution and were analyzed through one survey run (10 sweeps) and three main runs (peak jumping, 40 sweeps). Results are expressed as µg Cu per mg wet liver weight and as total ng per mitochondrial mass. Daily autotuning optimized instrument performance, ensuring adherence to the manufacturer’s specifications. Elemental analysis was performed at the Northwestern University Quantitative Bio-element Imaging Center (see Supplementary Table 2 for all settings).

### Estimation of mitochondrial fission and fusion

Using validated morphometric thresholds associated with mitochondrial fission and fusion states^43^, individual mitochondria were classified based on the distributions of the perimeter-to-area ratio and solidity. Cutoff values were derived using nonparametric tolerance interval analysis implemented in JMP (SAS Institute). For each parameter, a one-sided upper-tolerance interval was calculated from the empirical distribution, with 90% population coverage at 95% confidence (1–α = 0.95). The resulting upper tolerance limits were used as thresholds to identify mitochondria with extreme morphological values. This analysis yielded cutoff values of 0.01348 for perimeter-to-area ratio and 0.9905 for solidity. Mitochondria exceeding these thresholds were classified as high risk for fission (perimeter/area > 0.01348) or high risk for fusion (solidity > 0.9905), whereas mitochondria below these limits were classified as not at risk for dynamic remodeling. These thresholds were applied uniformly across sexes and treatment groups.

### Mitochondrial enzymatic or carrier activities, and mitochondrial ROS production

Complex and citrate synthase activities, along with ROS production, were assessed with minor modifications as detailed previously^72^. The data were analyzed for potential interactions using JMP Pro software. In JMP, the least-squares fitting procedure for a full factorial design fit a general linear model (GLM) that included all specified main effects (e.g., sex, treatment) and their interactions (e.g., sex × treatment). The model was estimated using the least-squares method, which minimized the sum of squared differences between observed and predicted values. ANOVA *p* values were obtained from F-tests that compared the variance explained by each effect (main or interaction) to the residual variance. This method enabled assessment of individual factor effects and their combined interactions, providing a comprehensive test of the full factorial structure. To further examine significant effects, a Tukey HSD (honestly significant difference) all-pairs post hoc test was performed, adjusting for multiple comparisons when analyzing group means. All analyses were conducted in JMP 18 using the Standard Least Squares platform with all model effects included.

### Lipid mediator, steroid hormone, and bile acid profiling

Liver tissue and mitochondrial pellets were analyzed for oxylipins, steroid hormones, and bile acids using targeted UPLC-MS/MS with isotope dilution quantification. Samples were homogenized in organic solvents containing deuterated internal standards, centrifuged, filtered, and analyzed on QTRAP mass spectrometers under scheduled multiple-reaction monitoring conditions. For liver analysis, approximately 50 mg of tissue was weighed into a 2 mL polypropylene Eppendorf tube with three 3 mm stainless steel balls. The sample was then mixed with 5 µL of 0.2 mg/mL butylated hydroxytoluene/EDTA, 10 µL of a mix of 14 deuterated steroids and bile acids, 10 µL of a mix of 19 deuterated oxylipins, 10 µL of a 5 µM solution of CUDA/PUHA in 1:1 acetonitrile/methanol, and 215 µL of 1:1 acetonitrile/methanol to reach a final volume of 250 µL. The samples were then homogenized on a vertical ball mill (GenoGrinder 2010, Spec Sample Prep, Metuchen NJ) for 2 min at 1500 rpm. The homogenized sample was subsequently centrifuged for 5 min at 15,000 g at room temperature, chilled at − 20 °C for 20 min, and the supernatant filtered through a 0.2 μm PVDF filter in a 96-well format prior to analysis.

Oxylipins were separated using a 2.1 mm x 150 mm, 1.7 μm Acquity BEH C18 column (Waters, Milford, MA), while steroids were separated on a 2.1 mm x 100 mm, 1.7 μm Acquity BEH C18 column (Waters, Milford, MA). Samples were analyzed with a Nexera X2 liquid chromatograph (Shimadzu Corporation, Kyoto, Japan) and detected by electrospray ionization coupled with scheduled multiple reaction monitoring on a 6500 QTRAP (Sciex; Redwood City, CA), as previously described^72^. Quantification was performed using isotope dilution and internal standard ratio-response methods, with a 5-7-point calibration curve covering the reported concentrations. F2-isoprostanes (F2-IsoP) were identified as a complex cluster of analytes sharing the same mass transition as PGF2a. The F2-IsoP concentration was estimated based on the response of prostaglandin F2a (353.3 > 193.2 m/z), as previously detailed^41^.

### Statistical analysis

Statistical analyses were performed using JMP 18 (SAS Institute). For outcomes in which sex and treatment were evaluated simultaneously, two-way factorial ANOVA was used with sex and treatment as fixed factors and their interaction (sex × treatment) included in the model. Models were fitted using least-squares estimation in the JMP Fit Model platform, which accommodates slightly unbalanced group sizes. Post hoc comparisons were conducted using Tukey’s honestly significant difference (HSD) test.

Depending on the dataset, additional analyses included Student’s t-tests, one-way ANOVA, mixed-effects models (with mouse ID included as a random factor where appropriate), and least-squares means contrasts. Corresponding post hoc comparisons were performed using Tukey’s HSD or Johnson–Sidak–Bonferroni (SB) adjustments as appropriate.

Data are presented as mean ± standard deviation (SD) unless otherwise indicated; least-squares means ± standard error (SE) are reported for factorial ANOVA models. Statistical significance was defined as *P* < 0.05. For pathway-based bile acid visualizations, trends (*P* < 0.15) were displayed as indicated in the corresponding figure legends.

## Supplementary Information

Below is the link to the electronic supplementary material.

Supplementary Material 1

## Data Availability

All data supporting the findings of this study are available within the paper and its Supplementary Information .

## Declarations

### Artificial intelligence

Artificial intelligence technology was used to improve readability and language (Grammarly).
