# Supplementary material for: Sex-specific mitochondrial dysregulation and metformin response in Wilson disease
Source: Sci Rep. 2026 Jun 27;16:29570. doi: 10.1038/s41598-026-58720-7 (PMC13597304; doi:10.1038/s41598-026-58720-7)
Supplement: Supplementary file 1 [file 41598_2026_58720_MOESM1_ESM.pdf]

Supplementary Material

Sex-Specific Mitochondrial Dysregulation and Metformin Response in Wilson Disease

This supplementary file contains additional tables and figures supporting the main manuscript. The materials provide detailed methodological parameters, quantitative analyses, ultrastructural imaging, and literature context related to mitochondrial morphology, bioenergetic function, lipid metabolism, inflammation, and sex-specific responses to metformin treatment in a murine model of Wilson disease.

Contents

Supplementary Tables

Supplementary Table 1. ***Mouse identification, sex, treatment group, transmission electron microscopy (TEM) image counts, and summary of mitochondrial analyses.***

Supplementary Table 2. ***Inductively coupled plasma mass spectrometry (ICP-MS) instrumental settings, acquisition parameters, and operating conditions used for elemental analysis.***

Supplementary Table 3. ***Morphometric parameters and quantitative metrics used to assess mitochondrial shape and ultrastructure from TEM images.***

Supplementary Table 4. ***Summary of sex-specific effects of metformin reported in human studies across metabolic, inflammatory, cardiovascular, neurological, and oncological conditions.***

Supplementary Table 5. ***Summary of sex-specific effects of metformin reported in murine studies across various conditions.***

Supplementary Figures

Supplementary Figure 1. ***Ultrastructural features of hepatic mitochondria in Atp7b$^{-/-}$ mice.***

Supplementary Figure 2. ***Mitochondrial filamentous aggregates in male and female untreated and metformin-treated Atp7b$^{-/-}$ mice.***

Supplementary Figure 3. ***Mitochondrial respiratory chain Complex activities and hepatic lipid vacuole perimeter in Atp7b$^{-/-}$ mice.***

Supplementary Figure 4. ***Lipoxygenase (LOX)- and cyclooxygenase (COX)-derived oxylipin products identified during CPT1 inhibition under inflammatory condition.***

Supplementary Table 1. ***Mouse identification, sex, treatment group, transmission electron microscopy (TEM) image counts, and summary of mitochondrial analyses.***
The table lists individual mouse identifiers, sex, treatment condition (untreated or metformin-treated), the number of TEM images acquired per mouse, and the corresponding number of mitochondria evaluated (shown in parentheses). The final row reports the total number of TEM images analyzed and the cumulative number of mitochondria assessed across all animals.

ID	Sex	Treatment	Number of TEM Images/mouse (number of mitochondria evaluated)
**C01A**	M	None	10 (126)
**C02A**	F	None	12 (157)
**C03A**	F	None	10 (189)
**C04A**	M	None	11 (165)
**C05A**	M	None	13 (187)
**C06A**	M	None	10 (109)
**C07A**	F	None	10 (115)
**C08A**	F	None	10 (143)
**Met01A**	M	Metformin	9 (169)
**Met02A**	F	Metformin	10 (154)
**Met03A**	F	Metformin	10 (160)
**Met04A**	M	Metformin	20 (246)
**Met05A**	M	Metformin	10 (173)
**Met06A**	M	Metformin	11 (157)
**Met07A**	M	Metformin	10 (169)
**Met08A**	F	Metformin	10 (128)
**Met09A**	F	Metformin	10 (129)
**Met10A**	F	Metformin	10 (148)
		Total	**196 (2824)**

Supplementary Table 2. ***Inductive coupled plasma mass spectrometry (ICP-MS) instrumental settings, acquisition parameters, and operating conditions used for elemental analysis.***
The table summarizes plasma conditions, sample introduction components, gas flows, collision/reaction cell settings, monitored mass-to-charge ratios (m/z), integration times, and tuning parameters. Elements were quantified using helium (He) collision gas mode, with germanium (Ge) and rhodium (Rh) used as internal standards. Instrument sensitivity was optimized using cobalt (Co), yttrium (Y), and thallium (Tl) tuning masses. Each sample was analyzed in triplicate with three sweeps per replicate.

Parameter	Setting
**Plasma RF Power nebulizer**	1550 W MicroMist (Glass Expansion, Melborne Australia)
**Cones**	Nickel
**Nebulizer gas flow**	0.98 L/min
**Makeup gas flow**	0.23L/min
**Spray chamber**	Scott type double pass spray chamber
**Spray chamber temperature**	2°C
**Gas mode**	He
**Collision/ reaction gas flow**	3 mL/min
**m/z Q1 and Q3**	55 (Mn); 56(Fe); 63 (Cu); 72 (Ge); 103 (Rh)
**Integration time**	0.5s (Mn); 0.3s (Fe; Cu, Ge; Rh)
**Replicates**	3
**Sample depth**	8.0 mm
**Sweeps**	3×40
**Tune maximum sensitivity on**	59→59 (Co); 89→89 (Y); 205→205 (Tl)

Supplementary Table 3. ***Morphometric parameters and quantitative metrics used to assess mitochondrial shape and ultrastructure from transmission electron microscopy (TEM) images.***
This table summarizes the two-dimensional (2D) morphometric parameters and three-dimensional (3D) shape estimates used to quantify mitochondrial size, shape, boundary complexity, and ultrastructural features from transmission electron microscopy (TEM) images. Parameters include area-, perimeter-, axis-, convexity-, circularity-, and skeleton-based metrics derived using ImageJ and associated plugins. Three-dimensional estimates of mitochondrial volume, surface area, surface-to-volume ratio, and sphericity were calculated using geometric approximations based on measured 2D dimensions, assuming an ellipsoidal shape and a constant thickness derived from the minor axis. Symbols, definitions, and calculation formulas are provided for each parameter to ensure reproducibility and facilitate comparison across studies.

**2D metrics**			
**Parameter**	**Symbol**	**Description**	**Calculation / Source**
**Area**	A	Cross-sectional area of a mitochondrion (nm^2)	Obtained from ImageJ (calibrated)
**Aspect Ratio**	B	Ratio of the major to minor axes of the best-fitting ellipse	B = L/O (ImageJ)
**Circularity**	C	Shape circularity (0–1). Approaches 1 for perfect circle, lower for irregular/elongated shapes	$C = 4\pi A / Q^2$ (ImageJ)
**Convex Hull Area**	E	Area of the smallest convex polygon enclosing the mitochondrion	Obtained from ImageJ
**Solidity**	U	Ratio of actual area to convex hull area	U = A/E (ImageJ)
**Convex Perimeter**	F	Perimeter of convex hull boundary	Obtained from ImageJ
**Convexity**	D	Degree to which shape approximates a convex boundary	D = F/Q
**Convex Radius**	G	Equivalent circle radius for convex hull area	$G = \mathrm{sqrt}(E/\pi)$
**Perimeter**	Q	Length of outer mitochondrial boundary (nm)	Obtained from ImageJ
**Perimeter-to-Area Ratio**	R	Shape complexity relative to size; higher values indicate more invaginations/branching	R = Q/A
**Major Axis**	L	Length of the best-fit ellipse's major axis (nm)	Obtained from ImageJ
**Minor Axis**	O	Length of the best-fit ellipse's minor axis (nm)	Obtained from ImageJ
**Roundness**	S	ImageJ Roundness: $4A/(\pi L^2)$. Equals 1 for perfect circle; decreases as shape elongates	$S = 4A/(\pi L^2)$ (ImageJ)
**Elongation**	I	Degree of lengthening; equivalent to aspect ratio	I = L/O = B
**Form Factor**	J	Inverse of circularity; higher = more irregular	$J = Q^2/(4\pi A)$

**Integrated Density**	K	Total pixel intensity within mitochondrion	K = M × Area_px (ImageJ: Mean Gray Value × Area in pixels)
**Mean Pixel Value**	M	Average pixel intensity within mitochondrion	Obtained from ImageJ
**Mitochondrion Total Length**	P	Skeleton length including branches (nm)	Obtained from ImageJ skeletonization (AnalyzeSkeleton/MorphoLibJ)
**Straightness**	H	Ratio of end-to-end distance to skeleton length (0–1). 1 = straight, <1 = more curved	H = end-to-end / P

**3D Estimates (assumption: thickness c = mean(O)/2)**			
**Parameter**	**Symbol**	**Description**	**Calculation / Source**
**Volume**	X	Estimated volume assuming ellipsoid with semi-axes a=L/2, b=O/2, c=mean(O)/2	X = (4/3)π a b c = (4/3)π (L/2)(O/2) c_avg
**Surface Area**	V	Estimated ellipsoid surface area (nm²) using Knud Thomsen approximation	V ≈ 4π ((a^p b^p + a^p c^p + b^p c^p)/3)^(1/p), p=1.6075
**Surface-to-Volume Ratio**	W	Surface area relative to volume	W = V / X
**Sphericity (2D proxy)**	T	Ratio of circle radius from convex area vs. ellipse minor axis; 1 for circle	T = G / O (report as '2D sphericity proxy')

Supplementary Table 4. ***Sex-specific differences in metformin responses reported in human clinical and epidemiological studies.***
This table summarizes published human clinical, cohort, randomized, and epidemiological studies reporting sex-specific differences in the effects of metformin across metabolic, cardiovascular, inflammatory, oncological, and infectious disease contexts. Information includes study design, treatment regimen, dose and route of administration, disease or outcome of interest, and reported sex differences in therapeutic efficacy, adverse drug reactions, or clinical outcomes. The table highlights the heterogeneity of sex-dependent responses to metformin and provides context for interpreting sex-specific effects observed in the present study.

Reference	Study Type	Treatment routine	Dose & Route	Disease & Variable of interest	Sex Differences in Effect of Metformin
Type 2 diabetes and insulin-resistance conditions					
Balkau et al. 2019 [1]	Cohort	5 y	As prescribed, in combination with other glucose lowering agents	Moderate to advanced chronic kidney disease with T2DM	Higher hypoglycemia frequency female vs males on combined treatments (59 vs 40%)
Carvalho et al. 2021 [2]	Cohort	As prescribed	As prescribed	Musculoskeletal pain in T2DM	Lower risk of musculoskeletal pain in both sexes; stronger effects in females
Crouse et al. 2020 [3]	Cohort	As prescribed	As prescribed	COVID-19 Mortality with T2DM	Lower risk of COVID-19 death in males but not females (OR 0.28; 95% CI 0.09–0.88; P=0.0286)
de Vries et al. 2020 [4]	Longitudinal Survey	12 m	As prescribed, oral	ADRs with T2DM	More ADRs in females, especially early in treatment
de Vries et al. 2024 [5]	Cohort	3-9 m after initiation of metformin	As prescribed, alongside alternate glucose-lowering drugs	HbA1C level after changes to other medications after initiation of metformin in T2DM	Slightly greater HbA1c reduction in males (7.6 to 6.7% vs 7.4 to 6.8% in females)
Esteghamati et al. 2013 [6]	Randomized clinical trial	12 wk	1000 mg/d, oral	Leptin & Omentin levels with T2DM	Reduced leptin in males and no changes in female
Lee et al. 2011[7]	Cohort	7 y	As prescribed, oral	CRC incidence with T2DM	Reduced CRC risk in both sexes; stronger in females
Bramante et al. 2021 [8]	Cohort	6 m	As prescribed, oral	COVID-19 Mortality with T2DM or Obesity	Reduced COVID-19 mortality in females; no change in males
Froldi 2024 [9]	Narrative Review	N/A	Variable, oral	Hyperglycemia, pharmacokinetics, ADRs, CRC-specific mortality,	Lower CRC-specific mortality, CRC incidence rate, COVID-19 severity,

				CRC incidence rate, COVID-19	COVID-19 mortality in females vs males. Higher ADRs in Females
Gulanski et al. 2023 [10]	Cohort	Baseline	1000-2000mg/kg/d, oral	Metabolic health and T2DM	Higher BMI, LDL-C & greater waist circumference & economic burden in females vs males with additional effects of race
Jiang et al. 2021 [11]	Cohort	30 d	As prescribed, oral	COVID-19, ARDS & T2DM	Lower ARDS incidence in females; no change in males
Lyons et al. 2013 [12]	Randomized trial	4 m	Titrated to $Hb_{A1C}\leq 7\%$	Cardiovascular metabolism in T2DM	Improved cardiac metabolism in females & worsened in males
Magee et al. 2015 [13]	Randomized Controlled Trial	As prescribed	As prescribed, alongside various drugs	Coronary Artery Disease in T2DM	Treatment as intensive but HbA1C & LDL-C targets less frequently met in females
Oktora et al. 2023 [14]	Cohort	As prescribed	As prescribed, alongside various drugs	T2DM	More medication switching & slower intensification of treatment in females
Raparelli et al. 2020 [15]	Cohort	As prescribed	As prescribed, oral, alongside GLP-1RA	Cardiovascular Events in T2DM	Reduced cardiovascular events for both sexes; stronger effect in females
Rodriguez & Johnson 2020 [16]	Cohort	As prescribed	As prescribed, oral	Mean Platelet Volume (MPV) in T2DM	Decreased MPV in both sexes; stronger effect in females
Wang et al. 2020 [17]	Meta-analysis	As prescribed	As prescribed, oral	CRC & mortality with T2DM	Reduced CRC mortality in both sexes; stronger effect in females
Other conditions					
Tang et al. 2019 [18]	Randomized Crossover	Single dose	1000 mg, oral	Food Effect & bioequivalence of drug products in healthy subjects	Higher metformin exposure in females vs males (likely due to lower body weight)
Wang et al. 2018 [19]	Epidemiological	As prescribed	As prescribed, oral	Myocardial Infarction	Higher myocardial infarction risk in males vs females

Abbreviations: T2DM, type 2 diabetes mellitus; ARDS, acute respiratory distress syndrome; CRC, colorectal cancer; ADRs, adverse drug reactions.

Supplementary Table 5. ***Sex-specific effects of metformin reported in murine and other rodent studies.*** This table summarizes published murine and other rodent studies reporting sex-specific differences in biological responses to metformin across metabolic, inflammatory, neurological, cardiovascular, oncological, and aging-related models. Information includes treatment duration, dose, and route of administration; disease model or physiological context; outcome measures; and reported sex-dependent effects of metformin. Together with Supplementary Table 4, this table provides translational context for interpreting sex-specific mitochondrial, metabolic, and inflammatory responses observed in the present study.

Reference	Treatment routine	Dose	Disease & Variable of interest	Sex Differences in Effect of Metformin
Type 2 diabetes and insulin-resistant conditions				
Ivić et al. 2023 [20]	13 wk	50 mg/kg/d, s.c.	Metabolic disease	Greater glucose tolerance after reproductive senescence in females vs. males. Protected muscle insulin sensitivity but larger hepatic lipid droplets in males
Lu et al. 2024 [21]	Gestational	300 mg/kg/d, oral (maternal gavage)	GDM effects on offspring	Normalized metabolic disease in males but no effect in females
Matić et al. 2021 [22]	From 20-65 wk of age	50 mg/kg/d, s.c. with high sugar & fat diet	Prediabetes, Obesity	Reduced Sod activity in males. Increased *Gpx1* expression in blood vessels in females.
Schoonejans et al. 2021 [23]	Gestational	300 mg/kg/d, Oral (maternal, dietary)	Obese glucose-intolerant pregnancy effect on offspring	Numerous increased metabolic risk factors with variable sex differences to specific factors
Silamikele et al. 2021 [24]	10 wk	50 mg/kg/d, drinking water	Gut microbiome in T2DM model	Pronounced gut microbiome changes; stronger effects in females
Other conditions				
De Angelis et al. 2022 [25]	7 d	200 mg/kg/d, i.p	Peripheral nerve lesion & chronic pain	Better recovery in males but not females
Gilbert et al. 2023 [26]	7 d	200 mg/kg/d, i.p.	Spinal Cord injury recovery	Reduced amoeboid microglia in males but not females
Inyang et al. 2019 [27]	7 d	200mg/kg/d, i.p.	Hyperalgesia responses to plantar incision	Reduced hypersensitivity & hyperalgesia priming in males but not females
Kuppusamy et al. 2024 [28]	5 d	150 mg/kg/d, i.v.	Cancer & chemotherapy-induced neuropathic pain (via bortezomib)	Reduced tumor growth & volume in females but not males
Anisimov et al. 2010 [29]	3-21 m	100 mg/kg/d, drinking water	Lifespan & tumor burden	Increased lifespan & reduced tumor malignancy rate in females but not males
McNair et al. 2023 [30]	4 wk	200 mg/kg/d, drinking water	Pulmonary hypertension-induced RV dysfunction	Protection from hypotrophy & restored RV function in males but not females

Quaile et al. 2010 [31]	13 wk	0, 200, 600, 900 or 1200 mg/kg/d, oral (gavage)	Drug toxicity & kinetics	Decreased b.w., increased liver enzymes, alopecia in males. Several deaths among females. Additional sex-specific shifts in blood electrolyte, urea & creatine levels
Anisimov et al. 2015 [32]	Neonatal	100 mg/kg/d, s.c.	Spontaneous tumorigenesis & lifespan	Greater lifespan & decreased b.w.in males. Increased b.w. in females
Aruldas et al 2023 [33]	Before cocaine treatment	200 mg/kg/d, i.p.	Cocaine sensitization	Various sex difference in specific protein levels
Ruddy et al 2019 [34]	Postnatal d 9-12, 21-27 or 50-56	20 mg/kg or 200 mg/kg/d, s.c or i.p.	Cognitive recovery from neonatal stroke	Improved stem cell pool & rescued cognitive deficits enabled by estradiol in normal females & supplemented males. Effects inhibited by testosterone in normal males or supplemented females
Salomaki-Myftari et al. 2016 [35]	Prenatal (0-18 d post mating for the mother)	300 mg/kg, maternal gavage	Metabolic & gut microbial responses in neuropeptide Y overexpression	Increased weight gain & serum insulin in females; opposite effects in males. Microbiome composition correlated with metabolic phenotype in males
Wolff et al. 2020 [36]	8 wk	1000 ppm, oral with rapamycin 16 ppm	Protein synthesis on rapamycin	Metformin reduced but did not eliminate sex differences in proteotoxic seen on rapamycin

Abbreviations: b.w. body weight; RV, right ventricular; GDM gestational diabetes mellitus.

Supplementary Figure 1. *Ultrastructural features of hepatic mitochondria in Atp7b$^{-/-}$ mice*

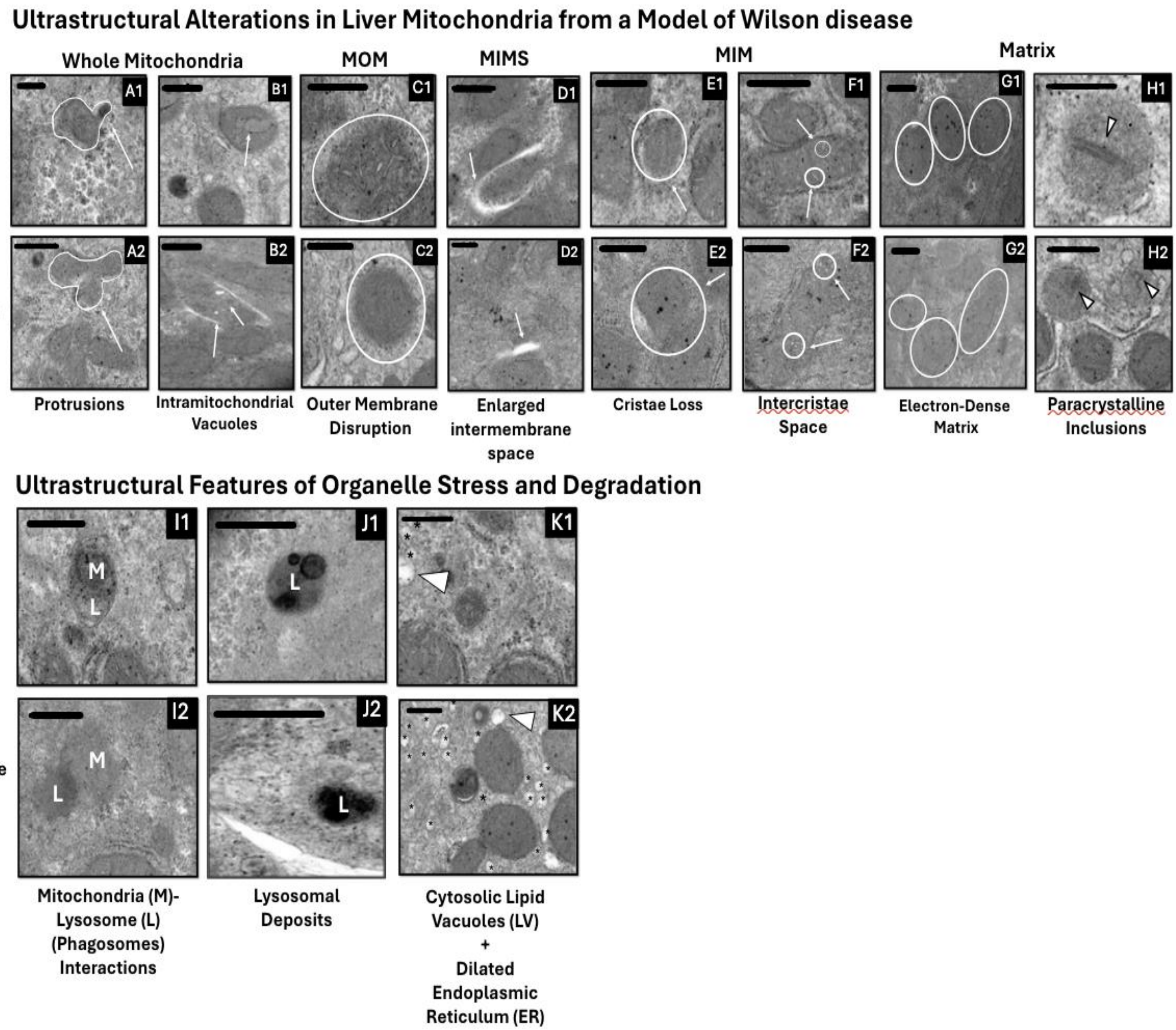

Representative transmission electron microscopy (TEM) images illustrating ultrastructural alterations in hepatocyte mitochondria from male and female Atp7b$^{-/-}$ mice, including the mitochondrial outer membrane (MOM), mitochondrial intermembrane space (MIMS), inner mitochondrial membrane (MIM), and matrix. Panels labeled "1" correspond to males and panels labeled "2" correspond to females.

A1–A2. Moderate, small, and asymmetrical mitochondrial protrusions or mitochondria-derived vesicles or MDV (white arrow pointing to the contour).

B1–B2. Intramitochondrial vacuoles (white arrow).

C1–C2. Disruption of the outer mitochondrial membrane (white circles).

D1–D2. Enlarged spaces outside the matrix appearing as electron-lucent areas (white arrows).

E1–E2. Mitochondria lacking cristae (white arrows within circles) or displaying asymmetrically packed cristae.

F1–F2. Enlarged intercristae spaces within the matrix (white arrows within circles).

G1–G2. Electron-dense mitochondrial matrix accompanied by electron-dense deposits (white circles).

H1–H2. Paracrystalline filamentous inclusions arranged as straight, parallel electron-dense lines (white arrow).
I1–I2. Engulfment of mitochondria (M) by lysosomes (L) within autophagosomes.
J1–J2. Electron-dense deposits within lysosomes (L).
K1–K2. Cytosolic lipid vacuoles (white arrowhead) and dilated endoplasmic reticulum (black asterisk).
Scale bar = 500 nm for all images.

Supplementary Figure 2. ***Mitochondrial filamentous aggregates in male and female untreated and metformin-treated*** $Atp7b^{-/-}$ ***mice***

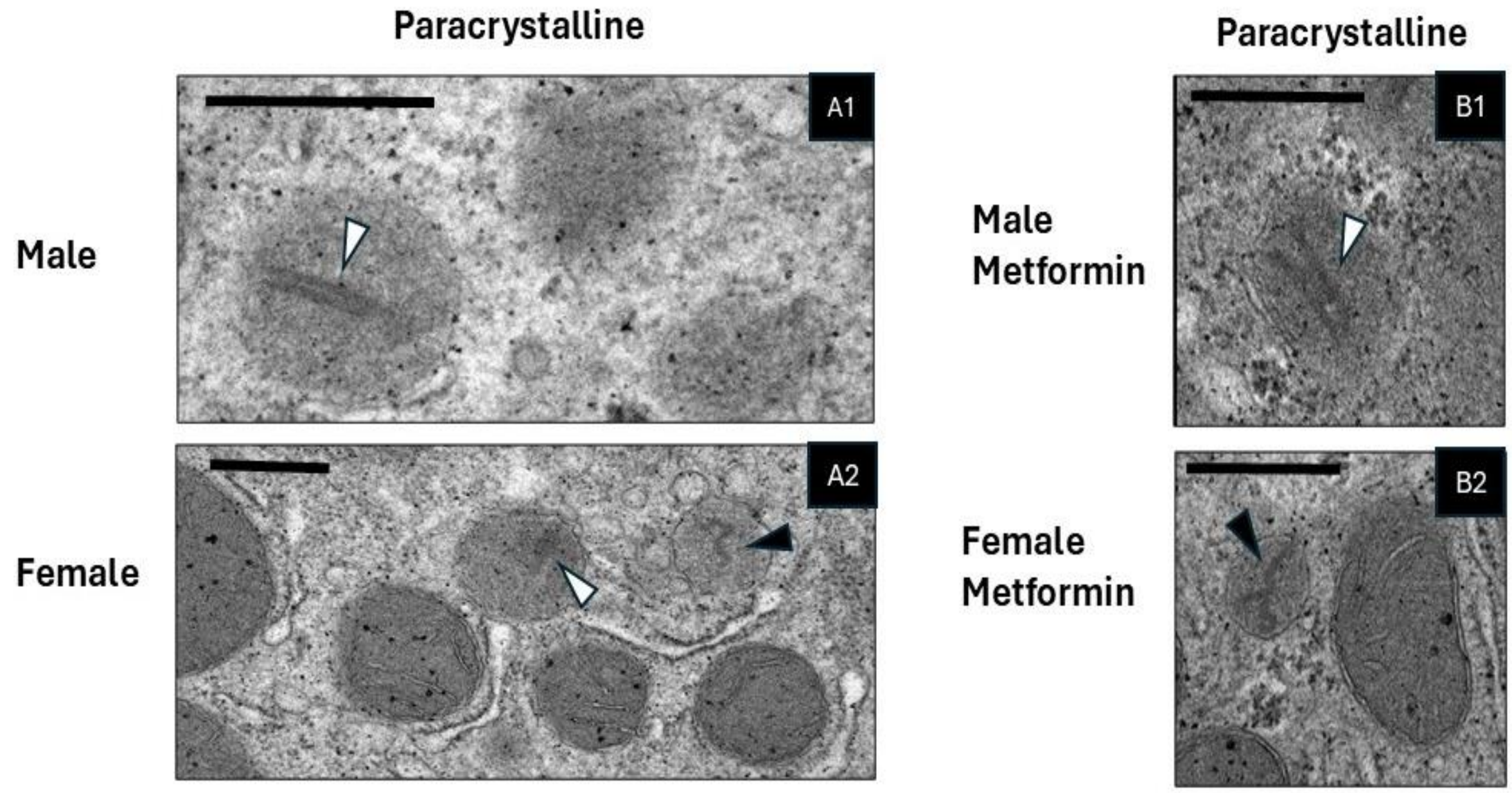

Representative transmission electron microscopy (TEM) images showing mitochondrial filamentous aggregates classified as paracrystalline inclusions[37-39] in hepatocytes from male and female mice in untreated and metformin-treated Wilson disease (WD) groups.

A1–A2; B1–B2. Paracrystalline filamentous inclusions arranged as straight, parallel, electron-dense lines (white arrows) or as short electron-dense rods (black arrows) within the mitochondrial matrix.

Quantitative analysis of TEM images showed that these inclusions occurred at low incidence across experimental groups. In untreated mice, males and females exhibited comparable proportions of mitochondria containing paracrystalline inclusions (0.68% vs 0.50%, respectively; χ^2 test, $P = 0.6795$), and crystalloid inclusions were not detected. Metformin treatment increased the prevalence of paracrystalline inclusions in both sexes (males: 2.74%, $P = 0.0048$ vs untreated males; females: 2.92%, $P = 0.0010$ vs untreated females). No significant sex difference was observed among metformin-treated mice.

Scale bar = 500 nm.

Supplementary Figure 3. ***Mitochondrial respiratory chain Complex activities and hepatic lipid vacuole perimeter in Atp7b$^{-/-}$ mice***

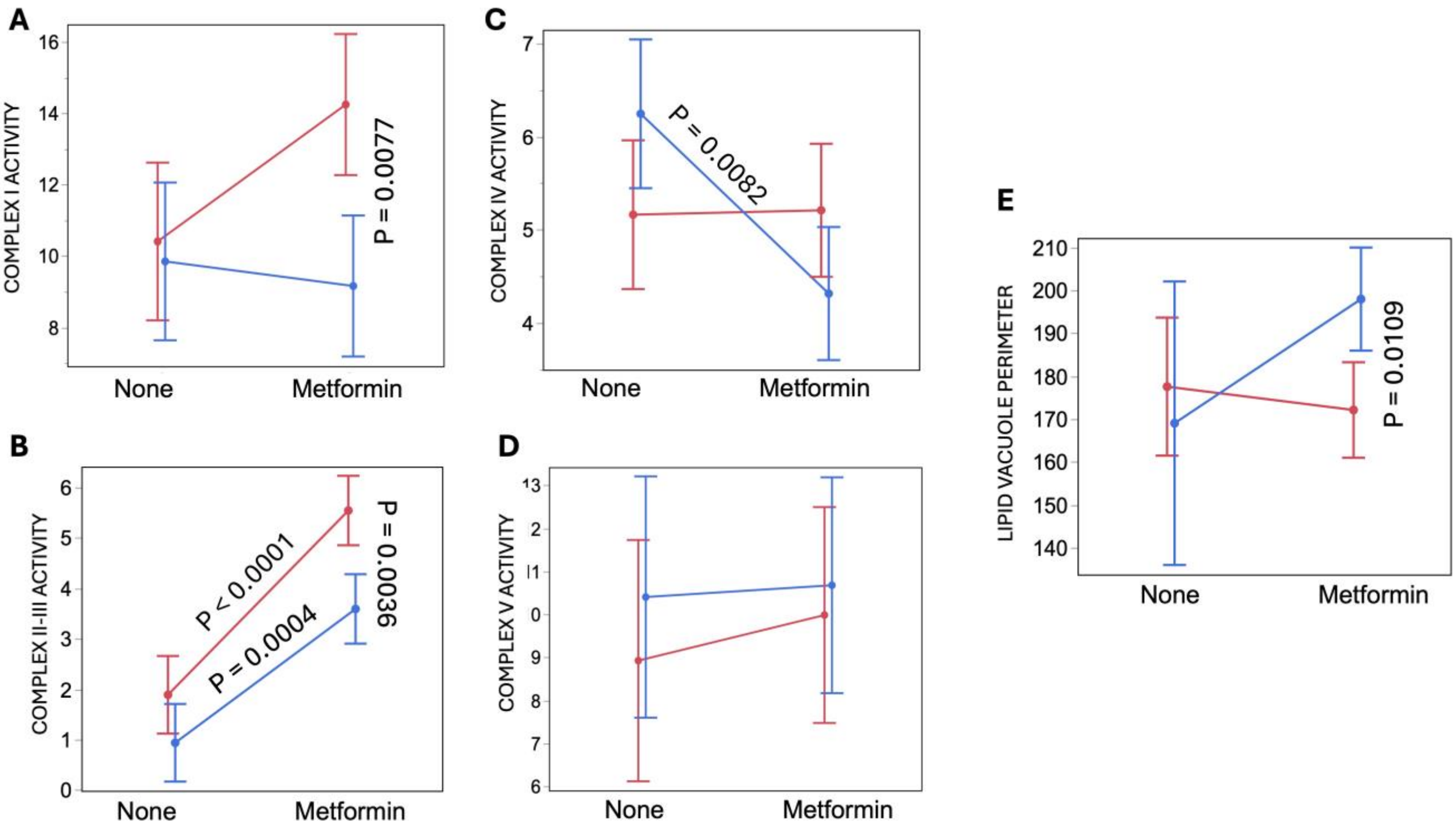

Complex I (A), Complex II–III (B), Complex IV (C), and Complex V (D) activities were measured in mitochondria-enriched liver fractions (n = 18 samples) and expressed as nmol × (min × mg protein)$^{-1}$. Panel E shows lipid vacuole perimeter expressed in nanometers (nm).
Data are presented as least-squares means ± standard error (SE) for each treatment group stratified by sex (females, red; males, blue). Statistical analysis was performed using a two-way factorial ANOVA with sex and treatment as fixed factors and their interaction (sex × treatment) included in the model. Post hoc comparisons were conducted using Tukey's honestly significant difference (HSD) test. Least-squares means were estimated using JMP 18 (SAS Institute).

Supplementary Figure 4. ***Lipoxygenase (LOX)- and cyclooxygenase (COX)-derived oxylipin products identified during CPT1 inhibition under inflammatory conditions.***

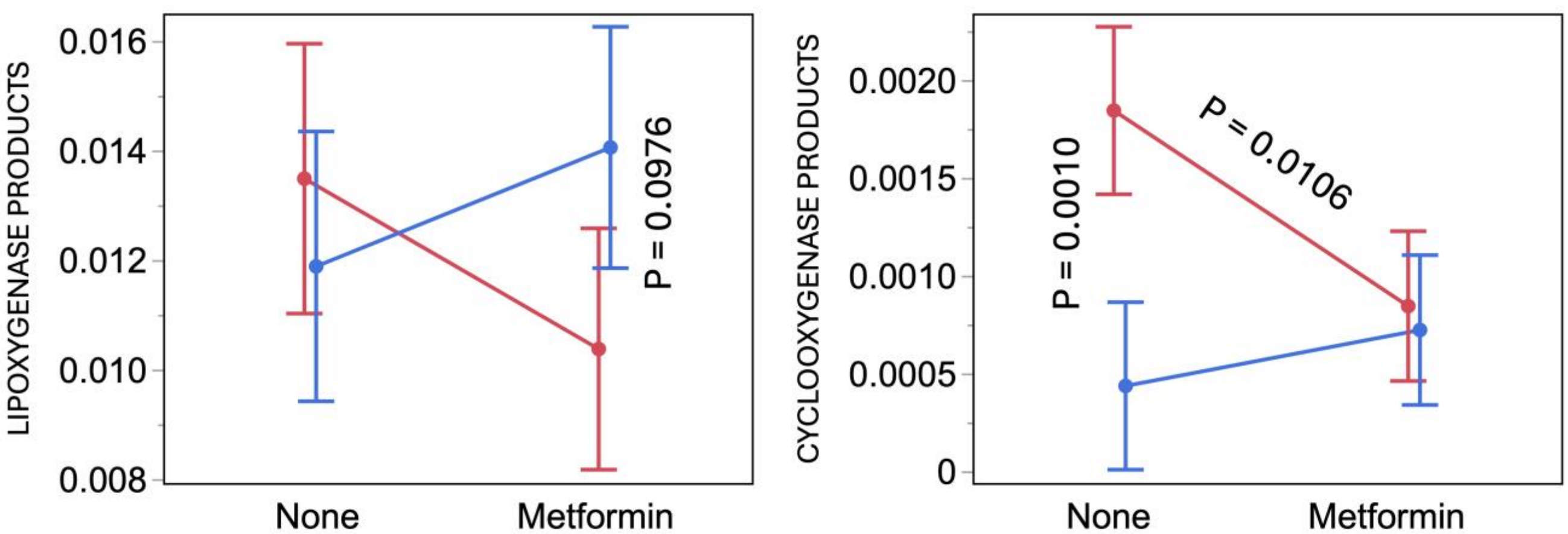

Hepatic lipoxygenase (LOX)-derived oxylipins overlapping with those reported by Misheva et al. [40] (including 11-HETE, 12-HEPE, and 12-HETE) showed a significant sex × treatment interaction ($P = 0.0295$), with a trend toward lower levels in metformin-treated females compared with males ($P = 0.0976$). Cyclooxygenase (COX)-derived oxylipins overlapping with the Misheva et al. study (PGF2α and TBX2) showed significant effects of sex ($P = 0.0012$) and a sex × treatment interaction ($P = 0.0044$). Untreated females exhibited higher levels than untreated males ($P = 0.010$), whereas metformin treatment significantly reduced these oxylipins in females ($P = 0.016$). Data are presented as least-squares means ± standard error (SE) for each treatment group stratified by sex (females, red; males, blue). Statistical analysis was performed using a two-way factorial ANOVA with sex and treatment as fixed factors and their interaction (sex × treatment) included in the model. Post hoc comparisons were conducted using Tukey's honestly significant difference (HSD) test. Least-squares means were estimated using JMP 18 (SAS Institute).

1 Balkau, B. *et al.* Impact of sex and glucose-lowering treatments on hypoglycaemic symptoms in people with type 2 diabetes and chronic kidney disease. The French Chronic Kidney Disease - Renal Epidemiology and Information Network (CKD-REIN) Study. *Diabetes Metab* **45**, 175–183, doi:10.1016/j.diabet.2018.03.007 (2019).
2 Carvalho, E. S. A. P., Harmer, A. R., Ferreira, M. L. & Ferreira, P. H. The effect of the anti-diabetic drug metformin on musculoskeletal pain: A cross-sectional study with 21,889 individuals from the UK biobank. *Eur J Pain* **25**, 1264–1273, doi:10.1002/ejp.1747 (2021).
3 Crouse, A. B. *et al.* Metformin Use Is Associated With Reduced Mortality in a Diverse Population With COVID-19 and Diabetes. *Front Endocrinol (Lausanne)* **11**, 600439, doi:10.3389/fendo.2020.600439 (2020).
4 de Vries, S. T., Denig, P., Ekhart, C., Mol, P. G. M. & van Puijenbroek, E. P. Sex Differences in Adverse Drug Reactions of Metformin: A Longitudinal Survey Study. *Drug Saf* **43**, 489–495, doi:10.1007/s40264-020-00913-8 (2020).
5 de Vries, S. T., de Vos, S., Oktora, M. P. & Denig, P. Sex disparities in medication changes after metformin initiation are associated with small, clinically non-relevant, sex differences in HbA1c levels during follow-up. *Pharmacoepidemiol Drug Saf* **33**, e5802, doi:10.1002/pds.5802 (2024).
6 Esteghamati, A. *et al.* Comparative effects of metformin and pioglitazone on omentin and leptin concentrations in patients with newly diagnosed diabetes: a randomized clinical trial. *Regul Pept* **182**, 1–6, doi:10.1016/j.regpep.2012.12.005 (2013).
7 Lee, M. S. *et al.* Type 2 diabetes increases and metformin reduces total, colorectal, liver and pancreatic cancer incidences in Taiwanese: a representative population prospective cohort study of 800,000 individuals. *BMC Cancer* **11**, 20, doi:10.1186/1471-2407-11-20 (2011).
8 Bramante, C. T. *et al.* Metformin and risk of mortality in patients hospitalised with COVID-19: a retrospective cohort analysis. *Lancet Healthy Longev* **2**, e34–e41, doi:10.1016/S2666-7568(20)30033-7 (2021).
9 Froldi, G. View on Metformin: Antidiabetic and Pleiotropic Effects, Pharmacokinetics, Side Effects, and Sex-Related Differences. *Pharmaceuticals (Basel)* **17**, doi:10.3390/ph17040478 (2024).
10 Gulanski, B. I. *et al.* Higher burden of cardiometabolic and socioeconomic risk factors in women with type 2 diabetes: an analysis of the Glycemic Reduction Approaches in Diabetes (GRADE) baseline cohort. *BMJ Open Diabetes Res Care* **11**, doi:10.1136/bmjdrc-2022-003159 (2023).
11 Jiang, N. *et al.* Association of metformin with mortality or ARDS in patients with COVID-19 and type 2 diabetes: A retrospective cohort study. *Diabetes Res Clin Pract* **173**, 108619, doi:10.1016/j.diabres.2020.108619 (2021).
12 Lyons, M. R. *et al.* Impact of sex on the heart's metabolic and functional responses to diabetic therapies. *Am J Physiol Heart Circ Physiol* **305**, H1584–1591, doi:10.1152/ajpheart.00420.2013 (2013).
13 Magee, M. F. *et al.* Sex, Prescribing Practices and Guideline Recommended, Blood Pressure, and LDL Cholesterol Targets at Baseline in the BARI 2D Trial. *Int J Endocrinol* **2015**, 610239, doi:10.1155/2015/610239 (2015).
14 Oktora, M. P., de Vos, S., de Vries, S. T., Hak, E. & Denig, P. Sex disparities in treatment patterns after metformin initiation among patients with type 2 diabetes mellitus. *Pharmacoepidemiol Drug Saf* **32**, 1395–1405, doi:10.1002/pds.5672 (2023).
15 Raparelli, V. *et al.* Sex Differences in Cardiovascular Effectiveness of Newer Glucose-Lowering Drugs Added to Metformin in Type 2 Diabetes Mellitus. *J Am Heart Assoc* **9**, e012940, doi:10.1161/JAHA.119.012940 (2020).
16 Rodriguez, B. A. T. & Johnson, A. D. Platelet Measurements and Type 2 Diabetes: Investigations in Two Population-Based Cohorts. *Front Cardiovasc Med* **7**, 118, doi:10.3389/fcvm.2020.00118 (2020).
17 Wang, Y., Xiao, J., Zhao, Y., Du, S. & Du, J. Effect of metformin on the mortality of colorectal cancer patients with T2DM: meta-analysis of sex differences. *Int J Colorectal Dis* **35**, 827–835, doi:10.1007/s00384-020-03539-5 (2020).
18 Tang, W., Engman, H., Zhu, Y., Dayton, B. & Boulton, D. W. Bioequivalence and Food Effect of Dapagliflozin/Saxagliptin/Metformin Extended-release Fixed-combination Drug Products Compared With Coadministration of the Individual Components in Healthy Subjects. *Clin Ther* **41**, 1545–1563, doi:10.1016/j.clinthera.2019.05.015 (2019).

19 Wang, S. H., Chen, W. J., Hsu, L. Y., Chien, K. L. & Wu, C. S. Use of Spontaneous Reporting Systems to Detect Host-Medication Interactions: Sex Differences in Oral Anti-Diabetic Drug-Associated Myocardial Infarction. *J Am Heart Assoc* **7**, e008959, doi:10.1161/JAHA.118.008959 (2018).
20 Ivic, V. *et al.* Elderly rats fed with a high-fat high-sucrose diet developed sex-dependent metabolic syndrome regardless of long-term metformin and liraglutide treatment. *Front Endocrinol (Lausanne)* **14**, 1181064, doi:10.3389/fendo.2023.1181064 (2023).
21 Lu, Y. *et al.* Progenies of gestational diabetes mellitus exhibit sex disparity in metabolism after respective therapies of insulin, glibenclamide, and metformin in dams during pregnancy. *Arch Physiol Biochem* **130**, 183–195, doi:10.1080/13813455.2021.1991957 (2024).
22 Matic, A. *et al.* Sex differences in oxidative stress level and antioxidative enzymes expression and activity in obese pre-diabetic elderly rats treated with metformin or liraglutide. *Croat Med J* **62**, 215–226, doi:10.3325/cmj.2021.62.215 (2021).
23 Schoonejans, J. M. *et al.* Maternal Metformin Intervention during Obese Glucose-Intolerant Pregnancy Affects Adiposity in Young Adult Mouse Offspring in a Sex-Specific Manner. *Int J Mol Sci* **22**, doi:10.3390/ijms22158104 (2021).
24 Silamikele, L. *et al.* Metformin Strongly Affects Gut Microbiome Composition in High-Fat Diet-Induced Type 2 Diabetes Mouse Model of Both Sexes. *Front Endocrinol (Lausanne)* **12**, 626359, doi:10.3389/fendo.2021.626359 (2021).
25 De Angelis, F. *et al.* Sex Differences in Neuropathy: The Paradigmatic Case of MetFormin. *Int J Mol Sci* **23**, doi:10.3390/ijms232314503 (2022).
26 Gilbert, E. A. B., Livingston, J., Garcia-Flores, E., Kehtari, T. & Morshead, C. M. Metformin Improves Functional Outcomes, Activates Neural Precursor Cells, and Modulates Microglia in a Sex-Dependent Manner After Spinal Cord Injury. *Stem Cells Transl Med* **12**, 415–428, doi:10.1093/stcltm/szad030 (2023).
27 Inyang, K. E. *et al.* Indirect AMP-Activated Protein Kinase Activators Prevent Incision-Induced Hyperalgesia and Block Hyperalgesic Priming, Whereas Positive Allosteric Modulators Block Only Priming in Mice. *J Pharmacol Exp Ther* **371**, 138–150, doi:10.1124/jpet.119.258400 (2019).
28 Kuppusamy, P., Haque, M. M., Traub, R. J. & Melemedjian, O. K. Targeting metabolic pathways alleviates bortezomib-induced neuropathic pain without compromising anticancer efficacy in a sex-specific manner. *Front Pain Res (Lausanne)* **5**, 1424348, doi:10.3389/fpain.2024.1424348 (2024).
29 Anisimov, V. N. *et al.* Gender differences in metformin effect on aging, life span and spontaneous tumorigenesis in 129/Sv mice. *Aging (Albany NY)* **2**, 945–958, doi:10.18632/aging.100245 (2010).
30 McNair, B. D. *et al.* Metformin protects against pulmonary hypertension-induced right ventricular dysfunction in an age- and sex-specific manner independent of cardiac AMPK. *Am J Physiol Heart Circ Physiol* **325**, H278–H292, doi:10.1152/ajpheart.00124.2023 (2023).
31 Quaile, M. P. *et al.* Toxicity and toxicokinetics of metformin in rats. *Toxicol Appl Pharmacol* **243**, 340–347, doi:10.1016/j.taap.2009.11.026 (2010).
32 Anisimov, V. N. *et al.* Sex differences in aging, life span and spontaneous tumorigenesis in 129/Sv mice neonatally exposed to metformin. *Cell Cycle* **14**, 46–55, doi:10.4161/15384101.2014.973308 (2015).
33 Aruldas, R., Orenstein, L. B. & Spencer, S. Metformin Prevents Cocaine Sensitization: Involvement of Adenosine Monophosphate-Activated Protein Kinase Trafficking between Subcellular Compartments in the Corticostriatal Reward Circuit. *Int J Mol Sci* **24**, doi:10.3390/ijms242316859 (2023).
34 Ruddy, R. M., Adams, K. V. & Morshead, C. M. Age- and sex-dependent effects of metformin on neural precursor cells and cognitive recovery in a model of neonatal stroke. *Sci Adv* **5**, eaax1912, doi:10.1126/sciadv.aax1912 (2019).
35 Salomaki-Myftari, H. *et al.* Neuropeptide Y Overexpressing Female and Male Mice Show Divergent Metabolic but Not Gut Microbial Responses to Prenatal Metformin Exposure. *PLoS One* **11**, e0163805, doi:10.1371/journal.pone.0163805 (2016).
36 Wolff, C. A. *et al.* Sex differences in changes of protein synthesis with rapamycin treatment are minimized when metformin is added to rapamycin. *Geroscience* **43**, 809–828, doi:10.1007/s11357-020-00243-8 (2021).
37 Caldwell, S. H. *et al.* Intramitochondrial crystalline inclusions in nonalcoholic steatohepatitis. *Hepatology* **49**, 1888–1895, doi:10.1002/hep.22851 (2009).

38 Ruffolo, R. & Covington, H. Matrix inclusion bodies in the mitochondria of the human liver: evidence of hepatocellular injury. *Am J Pathol* **51**, 101–116 (1967).
39 Mugnaini, E. Filamentous Inclusions in the Matrix of Mitochondria from Human Livers. *J Ultrastruct Res* **11**, 525–544, doi:10.1016/s0022-5320(64)80080-0 (1964).
40 Misheva, M. *et al.* Oxylipin metabolism is controlled by mitochondrial beta-oxidation during bacterial inflammation. *Nat Commun* **13**, 139, doi:10.1038/s41467-021-27766-8 (2022).
